# Ultrafast optical modification of exchange interactions in iron oxides

**DOI:** 10.1038/ncomms9190

**Published:** 2015-09-16

**Authors:** R.V. Mikhaylovskiy, E. Hendry, A. Secchi, J.H. Mentink, M. Eckstein, A. Wu, R.V. Pisarev, V.V. Kruglyak, M.I. Katsnelson, Th. Rasing, A.V. Kimel

**Affiliations:** 1 School of Physics, University of Exeter, Stocker Road, Exeter EX4 4QL, UK; 2 Radboud University Nijmegen, Institute for Molecules and Materials, Heyendaalseweg 135, 6525 AJ, Nijmegen, The Netherlands; 3 University of Hamburg, Center for Free-Electron Laser Science, Luruper Chaussee 149, 22761 Hamburg, Germany; 4 Shanghai Institute of Ceramics, Chinese Academy of Sciences, Shanghai 200050, China; 5 Ioffe Physical-Technical Institute, Russian Academy of Sciences, 194021 St Petersburg, Russia

## Abstract

Ultrafast non-thermal manipulation of magnetization by light relies on either indirect coupling of the electric field component of the light with spins via spin-orbit interaction or direct coupling between the magnetic field component and spins. Here we propose a scenario for coupling between the electric field of light and spins via optical modification of the exchange interaction, one of the strongest quantum effects with strength of 10^3^ Tesla. We demonstrate that this isotropic opto-magnetic effect, which can be called inverse magneto-refraction, is allowed in a material of any symmetry. Its existence is corroborated by the experimental observation of terahertz emission by spin resonances optically excited in a broad class of iron oxides with a canted spin configuration. From its strength we estimate that a sub-picosecond modification of the exchange interaction by laser pulses with fluence of about 1 mJ cm^−2^ acts as a pulsed effective magnetic field of 0.01 Tesla.

## Abstract (web summary)

In the ultrafast manipulation of magnetization by light, it is thought that the electric field couples only indirectly to spins via spin-orbit interaction. Here, the authors demonstrate inverse magneto-refraction, a direct optical modification of the exchange interaction allowed for any material symmetry.

## Graphical abstract

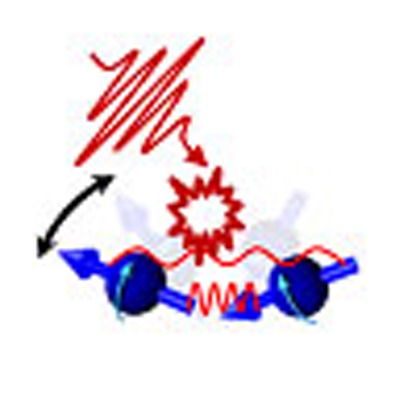

The symmetric part of the exchange interaction between spins is responsible for the very existence of magnetic ordering^1^. It is described by the Hamiltonian 
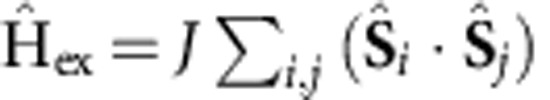
, where *J* is the exchange integral; 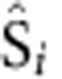 and 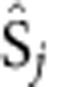 are the spins of the *i*th and *j*th adjacent magnetic ions. The antisymmetric part 
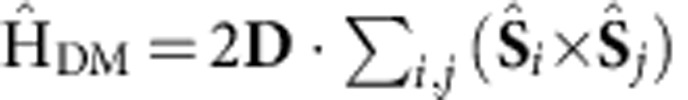
, characterized by a vector parameter **D** and called Dzyaloshinskii–Moriya interaction, gives rise to canted antiferromagnetism^2^,^3^ in iron oxides.

The ability to control the exchange interaction by light has intrigued researchers in many areas of physics, ranging from quantum computing^4^,^5^,^6^ to strongly correlated materials^7^,^8^,^9^. Laser-induced heating^10^,^11^ and photo-doping^9^,^12^ have been suggested to cause a modification of the exchange interaction. However, these phenomena rely on the absorption of light and are neither universal, that is, they are only present in specific materials, nor direct, that is, not instantaneous. Recently the time-resolved evolution of the exchange splitting in magnetic metals Ni and Gd subjected to ultrafast laser excitation was measured using photoelectron spectroscopy^13^ and angle-resolved photoemission^14^ techniques, respectively. Both of these techniques, unfortunately, do not allow to distinguish the intrinsic dynamics of the exchange parameters such as *J* from the demagnetization dynamics. Nevertheless, a direct, truly ultrafast effect of the electric field of light on the exchange interaction must be feasible in any material. In a medium of arbitrary symmetry, such an effect may be expressed phenomenologically by introducing an isotropic term in the Hamiltonian of the two-photon interaction between the light and spins


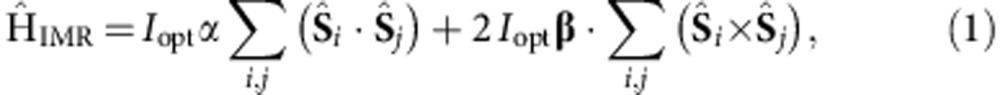


where *I*_opt_ is the intensity of light; *α* and **β** are some scalar and vector coefficients, respectively. The presence of the interaction Hamiltonian (1) manifests itself as a magnetic refraction, described by an isotropic contribution to the dielectric permittivity *ɛ*_IMR_∼*M*^2^ that leads to a dependence of the refractive index on the magnitude of the magnetization *M*^15^,^16^. The first term in the Hamiltonian describes the intensity dependent contribution, Δ*J*=*αI*_opt_, to the symmetric Heisenberg exchange integral *J*, whereas the second term describes the intensity dependent contribution, Δ**D**=**β***I*_opt_, to the Dzyaloshinskii–Moriya vector **D**. Recently the effect of isotropic magneto-refraction has been used to probe d–f exchange in EuTe^17^. As for other magneto-optical phenomena, isotropic magneto-refraction must be connected with an inverse effect^18^ described by the same Hamiltonian (1), that is, the optical generation of a torque **T**_*i*_ acting on a spin **S**_*i*_ due to the light-induced perturbation of the exchange parameters


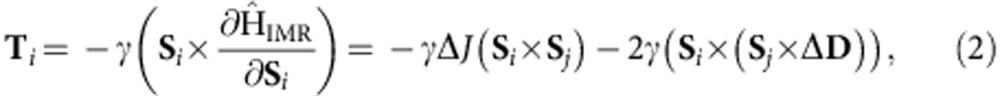


where γ is the absolute value of the gyromagnetic ratio. The torque (2) is zero in materials with collinear magnetic configurations since 
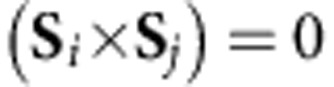
. In contrast to the torques exerted by the optical perturbation of the spin-orbit interaction^19^,^20^,^21^ or transient magnetic field^22^,^23^, it is independent of the light polarization.

In a broad class of transition metal oxides the magnetic order is governed by indirect exchange via ligand ions (superexchange)^1^ and is defined by virtual charge-transfer transitions of electrons between ligands and magnetic ions. Hence, one can anticipate the feasibility of a direct effect of the electric field of light on the exchange energy via virtual or real excitation of specific optical transitions that modify the hopping of the electrons between electronic orbitals centred at the transition metal ions and oxygen ligands, respectively.

Antiferromagnetic iron oxides possessing weak ferromagnetism, such as iron borate FeBO_3_, rare-earth orthoferrites RFeO_3_ (R stands for a rare-earth element) and hematite α-Fe_2_O_3_, are natural candidates for observing such ultrafast optical modification of the superexchange interactions. In these compounds the Fe^3+^ ions (spin quantum number *S*=5/2 and orbital momentum quantum number *L*=0) form two magnetic sublattices, the spins of which are antiferromagnetically coupled^24^. The presence of the Dzyaloshinskii–Moriya antisymmetric exchange interaction leads to a slight canting of the spins from the antiparallel orientation by an angle of ∼0.5–1°. The value of the canting is defined by the ratio *D/J* between the antisymmetric and symmetric exchange parameters. Thus, one could expect that an ultrafast optical perturbation of the exchange parameters could also change the ratio *D/J* and thereby trigger, by the torque defined in equation (2), the so-called quasi-antiferromagnetic resonance mode. This mode corresponds to oscillations of the magnitude of the weak magnetic moment without a change of its orientation^25^. According to equations (1) and (2), the ultrafast optical perturbation of the exchange parameters in these weak ferromagnets is an isotropic mechanism, that is, it can excite the quasi-antiferromagnetic resonance independently from the light polarization and propagation direction. The excited oscillating magnetic dipole in turn will lead to the generation of terahertz (THz) radiation which can be measured using terahertz emission spectroscopy^26^, as has been demonstrated before in experiments with ferromagnetic metals^27^,^28^,^29^ and antiferromagnetic insulators NiO^30^,^31^,^32^,^33^ and MnO^34^. In the present context, observation of THz emission due to laser excitation of the quasi-antiferromagnetic spin resonance via an isotropic mechanism would indicate an ultrafast manipulation of the exchange interactions. Importantly, to observe the THz radiation the emitting dipole must lie in the plane of the sample and therefore be perpendicular to the propagation direction of light^26^.

Here we reveal the inverse magneto-refractive effect to be responsible for ultrafast modulation of the superexchange interaction in a very broad class of canted antiferromagnets. Our findings are supported by a low-energy theory for the magnetic interactions between non-equilibrium electrons subjected to an external time-dependent electric field. We present quantitative estimates of the strength and timescale of the optical perturbation of the exchange parameters.

## Results

### THz emission from weak ferromagnets

Recently we reported measurements of THz emission signals in the rare-earth orthoferrites^26^ TmFeO_3_ and ErFeO_3_ which revealed the optical excitation of the high-frequency quasi-antiferromagnetic mode in these compounds along with the low-frequency quasi-ferromagnetic mode, another form of the antiferromagnetic resonance in canted antiferromagnets which involves the precession of the magnetization with no change in its length^25^. We also observed unexpected weak modes at ∼0.3 THz and assigned them to paramagnetic impurities^26^. The measurements suggested that the quasi-antiferromagnetic mode must be excited via a polarization-independent mechanism of coupling between light and spins. However, the data were not sufficient to identify the exact nature of the opto-magnetic excitation, in general, and to relate it to an optical perturbation of the exchange parameters *D*/*J* via inverse magneto-refractive effect, in particular.

**Figure 1:**
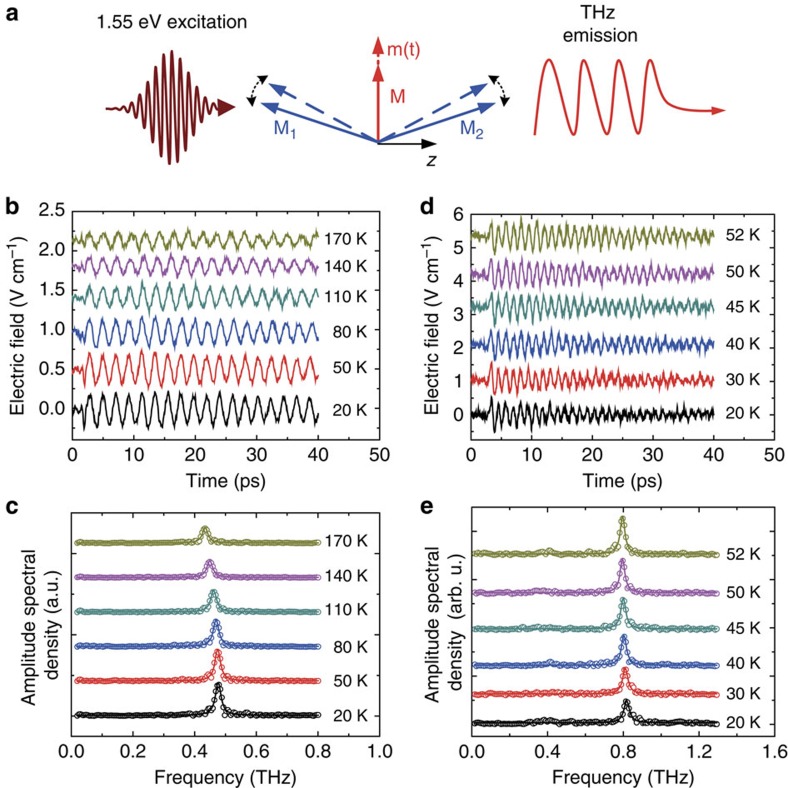
Terahertz emission generated in FeBO_3_ and TmFeO_3_. (**a**) The magnetization **M**=**M**_1_+**M**_2_ lies in the plane of the crystal sample plate. The optical pump is focused onto the sample plate along its normal (*z* axis), while the THz emission is collected along the same direction at the opposite side of the sample. The THz emission arises from the quasi-antiferromagnetic oscillations **m**(*t*). (**b**) The FeBO_3_ emission at different temperatures below 170 K. The zero time delay corresponds to an arbitrary starting position. The laser pulse arrives just before the commencement of the oscillations. (**c**) The spectra of the FeBO_3_ emission obtained from the data by Fourier transform (open circles) fitted by Lorentzian functions (solid lines). (**d**) The TmFeO_3_ emission at different temperatures below 55 K. (**e**) The TmFeO_3_ emission spectra (open circles) fitted with Lorentzian functions (solid lines).

To demonstrate the existence of the inverse magneto-refractive effect described above, and in particular the polarization-independent ultrafast optical perturbation of the exchange parameters *D*/*J*, we have studied the THz emission from a single FeBO_3_ cut perpendicularly to the *z*-crystallographic axis so that it lacks significant in-plane anisotropy of both optical and magnetic properties. The magnetization lying in the plane of the sample was aligned horizontally by a constant bias magnetic field of ∼0.1 T. The sample was illuminated by ∼100-fs laser pulses with their photon energy centred at 1.55 eV. We performed time-resolved detection of the THz radiation emitted from the sample in the direction of the *z* axis (see Fig. 1a). The waveforms generated at different temperatures are shown in Fig. 1b. We observe that the optical excitation of the sample leads to quasi-monochromatic emission at a frequency of ∼0.45 THz (Fig. 1c), which corresponds to the frequency of the quasi-antiferromagnetic mode in FeBO_3_ (ref. 35). The amplitude of the oscillations gradually decreases as the temperature approaches the Néel temperature *T*_*N*_∼350 K (see Supplementary Fig. 1).

**Figure 2:**
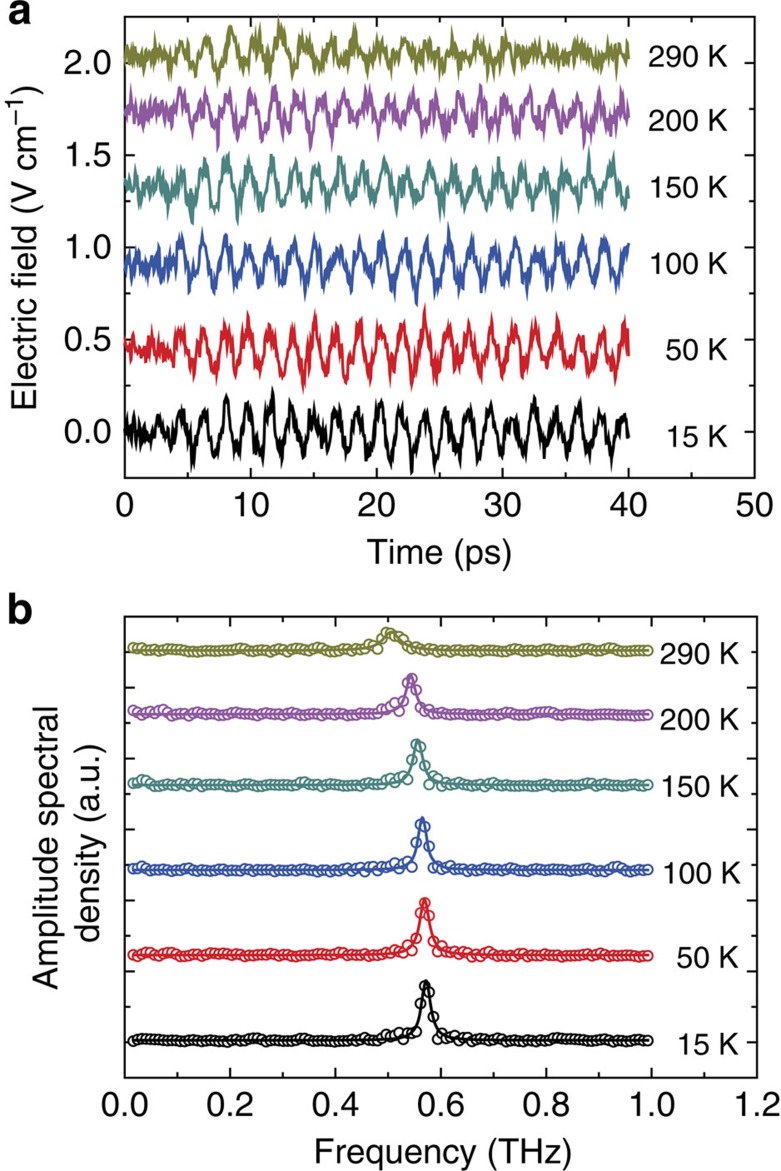
Terahertz emission generated in YFeO_3_. (**a**) The emission waveforms generated in the *x* cut YFeO_3_ sample at different temperatures. (**b**) The spectra of the YFeO_3_ emission obtained from the data by Fourier transform (open circles) fitted by Lorentzian functions (solid lines).

**Figure 3:**
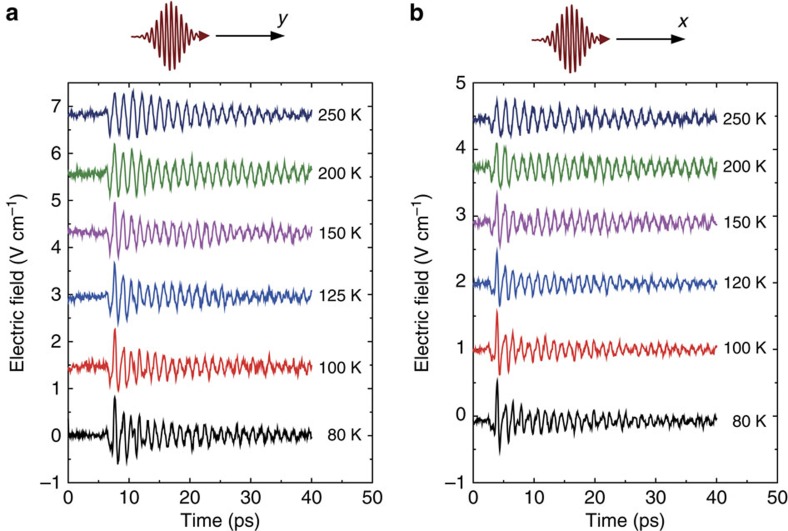
Terahertz emission generated in ErFeO_3_. (**a**) The ErFeO_3_ emission at different temperatures below room temperature generated by the optical pulses propagating along the *y* axis. (**b**) The ErFeO_3_ emission generated by the optical pulses propagating along the *x* axis.

To confirm that a similar mechanism is also present in other weak ferromagnets, we performed more detailed measurements of THz emission from orthoferrites similar to those reported in ref. 26, but for a temperature range in which only the quasi-antiferromagnetic mode was excited, making the interpretation of the experimental data less complex. Fig. 1e,d demonstrate that below 55 K the TmFeO_3_ single crystal plate cut perpendicularly to the *z*-crystallographic axis emits radiation with only one spectral component at the frequency of ∼0.8 THz, which is the frequency of the quasi-antiferromagnetic mode^25^ in TmFeO_3_ (see also Supplementary Fig. 1). To check that the observed effect is not due to the specific electronic structure of Tm^3+^ ions, we have performed similar experiments on the YFeO_3_ single crystal cut perpendicularly to the *x*-crystallographic axis (see Fig. 2). Fig. 2 shows that using an ultrafast optical excitation we are able to excite oscillations at a frequency of ∼0.55 THz, which again corresponds to the frequency of the quasi-antiferromagnetic mode in YFeO_3_ (ref. 25) (see also Supplementary Fig. 2). We have also observed similar polarization-insensitive ultrafast optical excitation of the quasi-antiferromagnetic mode in *y*- and *x*-cut samples of ErFeO_3_ (see Fig. 3), *x*-cut and *y*-cut DyFeO_3_ and in hematite α-Fe_2_O_3_ (see Supplementary Figs 3 and 4; Supplementary Note 1).

### Properties of the THz emission

**Figure 4:**
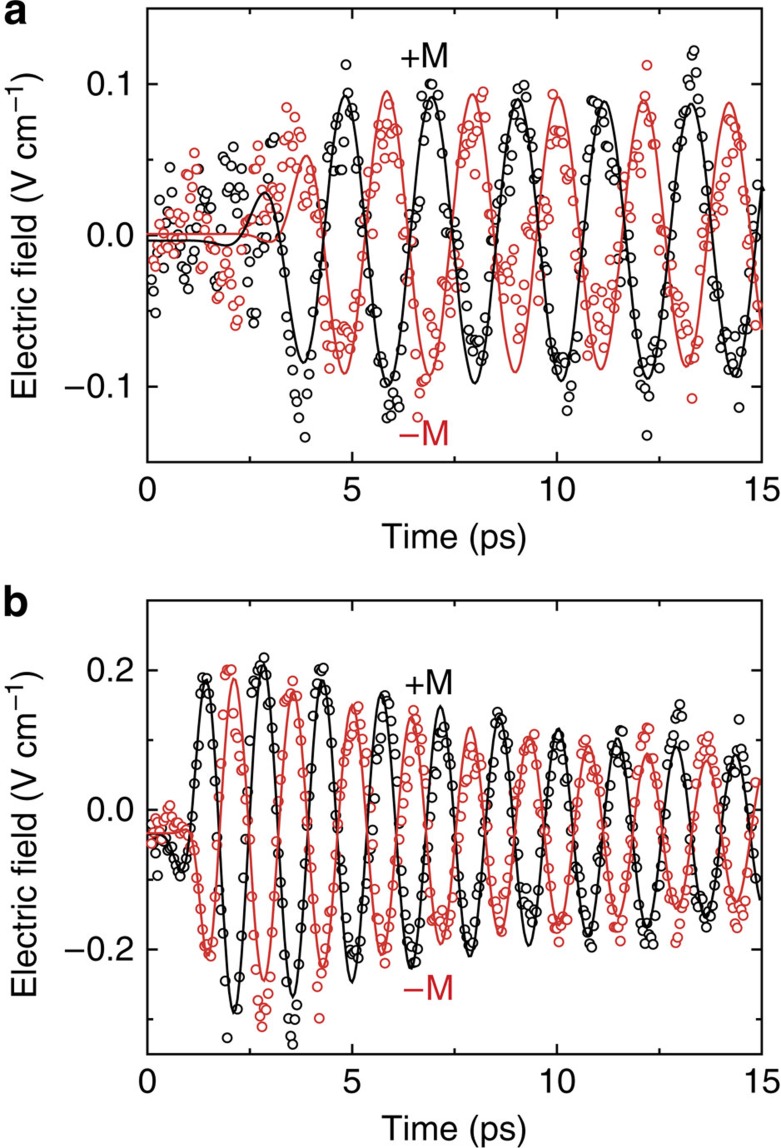
THz emission versus the magnetization direction in FeBO_3_ and ErFeO_3_. (**a**) The THz electric field emitted from the FeBO_3_ sample for opposite orientations of the magnetization (black and red open circles). (**b**) The THz electric field emitted from the *x* cut ErFeO_3_ sample for opposite orientations of the magnetization. The data are fitted with exponentially decaying sinusoids multiplied by error functions (solid lines).

To determine if the excitation mechanism is isotropic, we performed a set of measurements to systematically investigate its dependence on fluence and polarization of the laser pulse and found that the oscillation amplitudes depend linearly on the intensity of the pump (see Supplementary Figs 1 and 2) and are insensitive to the pump polarization (see Supplementary Fig. 5). By comparing the signals generated in the crystals pumped along different crystallographic directions, such as *y* and *x* axes in ErFeO_3_ (shown in Fig. 3) one can see that the excitation mechanism is isotropic with respect to the pump propagation direction as well. The phase of the measured oscillations changed by *π* with the reversal of the magnetization direction, confirming the magnetic origin of the signals (see Fig. 4). Moreover, this shows that the direction of the light-induced torque exciting the quasi-antiferromagnetic oscillations is determined by the orientation of the spins, and not by the polarization of light. All these observations are in perfect qualitative agreement with the anticipated features of an isotropic mechanism of optical modification of the exchange interaction described by equation (1).

## Discussion

The consistent observation of the photo-excitation of the quasi-antiferromagnetic mode in a range of compounds clearly indicates that this effect originates from the perturbation of the *D*/*J* ratio. The isotropic and polarization-insensitive character of the excitation rules out mechanisms based on the inverse Faraday effect^19^, which is sensitive to the ellipticity of the pump, or the inverse Cotton–Mouton effect^36^, which is sensitive to the polarization direction of the pump relative to the magnetization direction. We note that the THz emission observed from the antiferromagnets NiO (refs 30, 31, 32, 33) and MnO (ref. 34) did not contain a contribution that was isotropic relative to the pump polarization. Indeed the Dzyaloshinskii–Moriya antisymmetric exchange interaction is not allowed in these cubic insulators NiO and MnO and in the absence of an external magnetic field the torque (2) is equal to zero. Moreover, the observed effect cannot be attributed to the laser-induced change of the magneto-crystalline anisotropy as demonstrated in garnets^37^ and orthoferrites^38^,^39^ since this mechanism can trigger only the low-frequency quasi-ferromagnetic mode. This conclusion is further corroborated by the observation of this effect in FeBO_3_, which lacks significant in-plane anisotropy.

We would like to note that our demonstration of an ultrafast change of the ratio between the exchange parameters is based on the observation of the femtosecond excitation of the quasi-antiferromagnetic mode of spin resonance. Despite several optical pump–probe spectroscopy experiments on femtosecond laser excitation of spins in the orthoferrites and iron borate, the optical excitation of the quasi-antiferromagnetic mode has been very rarely observed. The very first observation of ultrafast laser excitation of both quasi-ferromagnetic and quasi-antiferromagnetic modes was reported for DyFeO_3_ in ref. 19 and later confirmed by Satoh *et al*.^40^ It was found, however, that for the chosen crystallographic orientation of the crystals the mechanisms of the excitation were dominated by the polarization dependent inverse Faraday and inverse Cotton–Mouton effects. Due to the fact that DyFeO_3_ was a strongly anisotropic material, discerning the helicity independent contribution from the data were not possible. Later studies only revealed the possibility of femtosecond helicity dependent excitation of the quasi-ferromagnetic mode in TmFeO_3_ (ref. 39), HoFeO_3_ (ref. 41), FeBO_3_ (ref. 36), ErFeO_3_ (ref. 42), and SmPrFeO_3_ (ref. 43). As a result of laser-induced heating and a subsequent spin-reorientation phase transition, an ultrafast excitation of again the quasi-ferromagnetic mode was reported for TmFeO_3_ (ref. 38), ErFeO_3_ (ref. 42) and SmFeO_3_ (ref. 44). No optically induced spin dynamics was reported for hematite.

The main reason why the isotropic, polarization-independent effect, reported here has not been observed before is that the detection in the aforementioned experiments was based on the magneto-optical Faraday effect which probes the spins indirectly that is, it strongly relies on the magneto-optical susceptibility and does not provide a direct picture of spin dynamics. Using THz emission spectroscopy, which is a more direct probe of the oscillating magnetization^26^, we have been able to identify the isotropic contribution to the optical excitation of the quasi-antiferromagnetic spin resonance, which is the principal result of this paper. We also point out that the excitation of the quasi-antiferromagnetic mode via the inverse Faraday effect is possible only in samples with the magnetization pointing out-of-plane. However in this geometry the THz waves cannot be emitted from the sample, hence we do not observe inverse Faraday-like effects in our THz signals.

**Figure 5:**
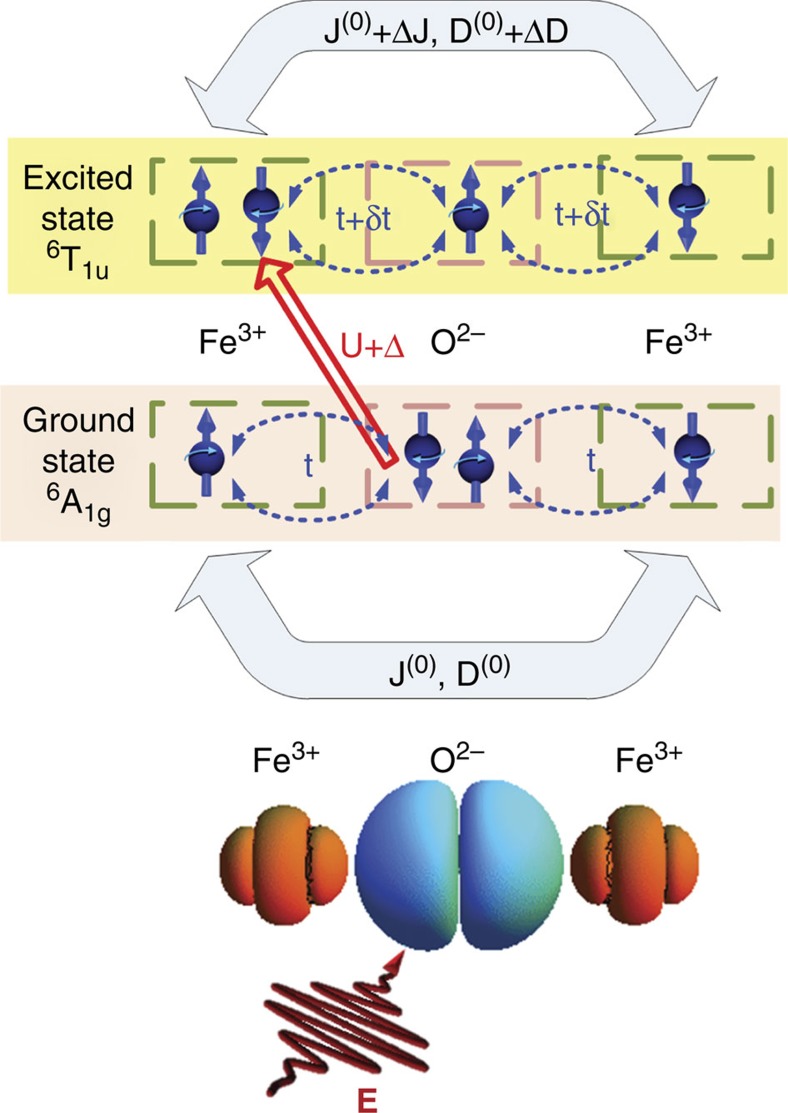
The mechanism for optical modulation of the exchange in iron oxides. In the ground state ^6^A_1g_ the exchange interactions *J*^(0)^ and *D*^(0)^ between the iron Fe^3+^ spins are mediated by the spins of the oxygen O^2−^ ions and occur due to the virtual hopping *t* (shown with dashed blue arrows) of electrons (blue balls with arrows schematically showing the ‘up' and ‘down' directions of their spins) within the iron-oxygen cluster. The strong electric field **E** of the laser pulse of arbitrary polarization excites virtual electric-dipole transitions from the ground state ^6^A_1g_ to the excited state ^6^T_1u_ over the energy gap *U*+Δ in the iron-oxygen cluster. The transitions involve the charge transfer (red arrow) from the oxygen to the iron site thereby changing the hopping amplitude *t* +*δt* and the electronic wave functions. As a result the exchange parameters are modified to *J*^(0)^+Δ*J* and *D*^(0)^+Δ*D*.

To specify the possible optical transitions responsible for our observations, we note that the dispersion of the refraction coefficient for all these compounds is dominated by the off-resonant susceptibilities related to the electric dipole allowed charge-transfer transitions between the 2*p* orbitals of oxygen and the 3*d* orbitals of the Fe^3+^ ions above 3 eV (refs 45, 46, 47). During the laser pulse duration and the time of optical decoherence, the collective electron wave-functions are coherent superpositions of the wave-functions of the ground and excited states. Such ultrafast modification of the wave-functions affects the exchange interaction between the spins of the neighbouring Fe^3+^ ions and thus changes the energy of the superexchange interaction (see Fig. 5). One can therefore expect that the observed effect of light on the exchange interaction is inherent to all magnetic materials, the magnetic order of which is governed by superexchange. However, only when the spins are canted, either by the Dzyaloshinskii–Moriya interaction or by an applied magnetic field, such an ultrafast change of the exchange interaction will lead to excitation of the antiferromagnetic resonance and the subsequent emission of THz radiation in accord with equation (2).

Our data are in excellent agreement with the phenomenology of equation (1) that gives the simplest and most plausible explanation. A possible microscopic scenario underpinning the phenomenology of our results can be understood in the framework of a recently developed formalism^48^ for microscopic magnetic interactions out of equilibrium (see Methods sections and Supplementary Note 2). To demonstrate the effect of a femtosecond laser pulse on the super-exchange interaction we numerically evaluated the time-dependent exchange for a 3-ion Fe^3+^–O^2−^–Fe^3+^ cluster, which is characterized by a strong on-site Coulomb interaction *U* on the Fe^3+^ ions, an energy level shift Δ between the Fe^3+^ and O^2−^ ions, and an equilibrium hopping amplitude *t*_0_ between Fe and O ions. For a small ratio *t*_0_/*U*, the leading-order expression for the equilibrium superexchange in this system reads^49^ 
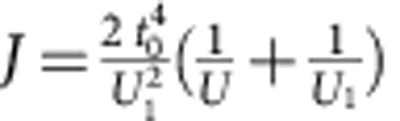
, where *U*_1_=*U+*Δ. By gradually switching on an oscillating off-resonant electric field we observe an enhancement of the exchange interaction proportional to the intensity of the laser pulse (see Supplementary Figs 6 and 7; Supplementary Note 2). To further understand the dependence of the superexchange on the laser field, we studied analytically a periodically driven cluster model. The shift of the energy levels under the periodic driving field can be understood within Floquet theory^50^ (see Supplementary Note 2), which gives an analytical expression for the change of the exchange interaction:


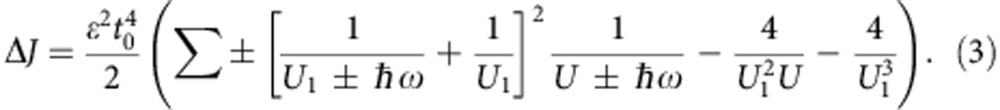


Here, *ɛ*=*eaE*_0_/*ħω* is the amplitude of the vector potential that describes the electric field in the Coulomb gauge with amplitude *E*_0,_ *e* and *a* are the unit charge and lattice constant, respectively, and *ω* is the frequency of the optical field. The terms dependent on±*ω* are the photon-assisted charge transfer excitations, while the last two terms describe a laser-induced decrease of the effective hopping amplitude within the Fe^3+^–O^2−^–Fe^3+^ cluster by a coherent destruction of tunnelling^51^. We obtain excellent quantitative agreement of Δ*J*/*J* between equation (3) and the numerical results obtained from the general theory (see Supplementary Note 2). In the experiment we typically have *ħω*∼*U*_1_/2, from which we conclude that the strengthening of the exchange interaction is caused by a photon-assisted charge-transfer excitation, as illustrated in Fig. 5. Using typical experimental parameters *U*=3 eV, Δ=0.25 eV, *t*_0_=0.5 eV and *ħω*=1.5 eV, we find that an optical pulse with a fluence of 1 mJ cm^−2^ and a corresponding electric field amplitude *E*_0_=0.12 V Å^−1^ should induce an increase of the exchange integral Δ*J*/*J* of over 1%. Our model analysis neither incorporates multi-orbital effects nor a description of the non-equilibrium Dzyaloshinskii–Moriya interaction, which certainly would be beyond the scope of this report. Importantly, we have shown theoretically that the optical manipulation of magnetic interactions is feasible already in the elementary super-exchange model defined by the Fe–O–Fe cluster.

**Figure 6:**
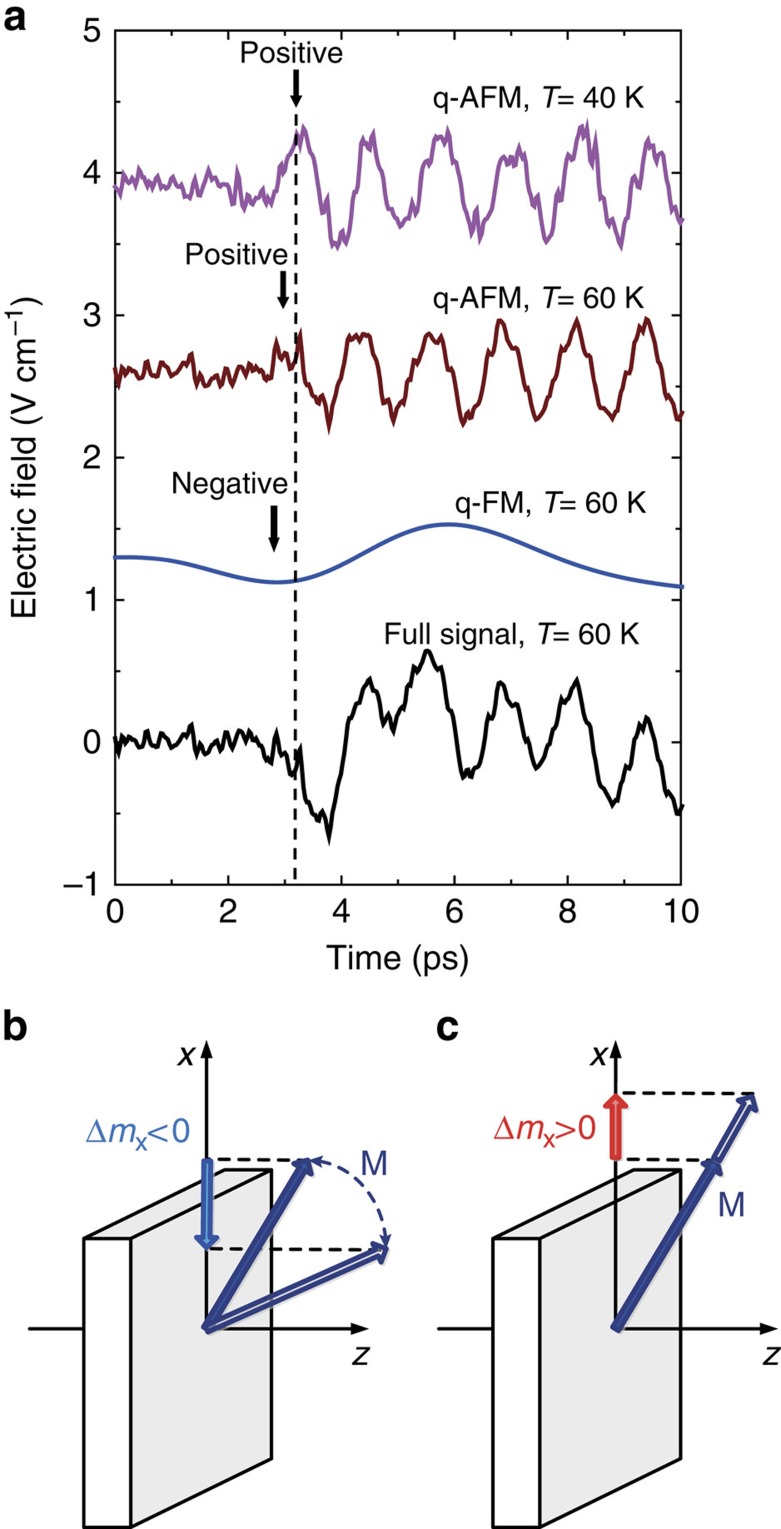
Determination of the absolute sign of the change of *D*/*J* in TmFeO_3_. (**a**) The signal emitted just below the spin reorientation temperature at 60 K (black) is shown together with its low-frequency (blue) and high-frequency part (brown). The latter part is in phase with the signal measured at 40 K which describes a quasi-antiferromagnetic (q-AFM) mode only (magenta). The low-frequency part corresponds to the quasi-ferromagnetic mode (q-FM). The first half-cycle of the quasi-antiferromagnetic mode has a different sign compared with the first half-cycle of the quasi-ferromagnetic mode (see dashed line). (**b**) During the spin reorientation the spin configuration of TmFeO_3_ continuously rotates in the (*xz*) plane, while keeping the weak ferromagnetic moment in the same plane. At low temperatures, the magnetization is oriented along the *x* axis. So, due to the laser-induced reorientation at 60 K, the *x*-component of the magnetization initially decreases. (**c**) Since the first half-cycle of the quasi-antiferromagnetic mode has a different sign, due to the exchange-driven torque the magnetization initially moves so as to have a positive *x*-component which implies an increase of *D*/*J.*

To determine whether laser excitation leads to a decrease or an increase of the ratio *D*/*J* we take advantage of the strong temperature dependence of the magnetic anisotropy, which is characteristic for many orthoferrites. For instance, heating of TmFeO_3_ from 80 to 90 K leads to a change of the equilibrium orientation of the weak magnetic moment from the *x* to the *z* axis. If the equilibrium orientation is changed as a result of a sudden heating by a femtosecond laser pulse, such a change is followed by oscillations of the weak magnetic moment in the (*xz*) plane at the frequency of the quasi-ferromagnetic mode (∼100 GHz)^38^,^39^. As discussed in ref. 26 in the range between 55 and 68 K, such low-frequency oscillations corresponding to the quasi-ferromagnetic mode are observed in THz emission spectra together with the high-frequency quasi-antiferromagnetic oscillations (see Fig. 6). We applied a low pass filter to the data (cutoff frequency 250 GHz) to isolate the quasi-ferromagnetic mode and a high-frequency filter (cutoff frequency 650 GHz) to isolate the quasi-antiferromagnetic mode. Such a choice of the cutoffs ensures the filtering out of the impurity modes which complicate the dynamics^26^. It is seen from Fig. 6 that the high-frequency mode measured at 60 K is in phase with that observed at 40 K. One can also see that the initial phases of the low-frequency quasi-ferromagnetic and high-frequency quasi-antiferrimagnetic modes are ∼180° apart. Note that for the *z*-cut TmFeO_3_ sample, with a net magnetic moment oriented upwards, a laser-induced spin-reorientation transition should trigger the quasi-ferromagnetic mode in such a way that the *M*_*x*_ component of the magnetization decreases. The observed difference in the phases between the two oscillations shows that the quasi-antiferromagnetic mode is triggered in such a way that the *M*_*x*_ component increases, which means that the canting angle becomes larger. Such a behaviour can only be explained by assuming that the quasi-antiferromagnetic oscillations are triggered by an increase of the ratio of the exchange parameters *D*/*J*. If this conclusion is true, in the *x*-cut sample the initial phases of the two modes must be the same, since the spin reorientation in this sample proceeds in the opposite direction. Measurements in the vicinity of the spin-reorientation temperature in ErFeO_3_ cut perpendicular to the *x* axis confirm this conclusion (see Supplementary Fig. 8; Supplementary Note 3). Interestingly, the increase of the ratio *D*/*J* cannot be explained on the basis of the simplistic model defined by the Fe–O–Fe cluster that predicts an increase of *J* and does not evaluate the change of *D*. However, the calculation of Δ*J* demonstrates the plausibility of the proposed mechanism of optical manipulation of the symmetric exchange interaction in principle.

To deduce the magnitude and timescale of the exchange modification from the experimental data, we have solved the Maxwell equations for a slab of a material with an oscillating magnetization triggered by a perturbation of the ratio *D/J* and calculated the electromagnetic radiation emitted by the slab into the free space. A quantitative analysis supports the sub-picosecond impact on the spin system (see Supplementary Fig. 9; Supplementary Notes 4 and 5). The absence of any significant broadband THz emission, which must accompany a laser-induced ultrafast demagnetization^27^,^28^ in iron borate and the orthoferrites (Fig. 1), supports the claim that femtosecond changes of the net magnetic moment can be neglected. The fact that the observed spin dynamics do not arise from the laser-induced heating is evidenced by the absence of a correlation between the strength of the observed signals and the specific heat and thermal conductivity of the studied materials. For example, the specific heat of YFeO_3_ below 100 K grows rapidly as the temperature increases while its thermal conductivity exhibits a pronounced peak around 30 K (ref. 52). At the same time the efficiency of the quasi-antiferromagnetic mode excitation in this compound does not depend on temperature at all (see Supplementary Fig. 2a). The observation of the very same effect of comparable strength in hematite with high optical absorption ∼2,000 cm^−1^ at 1.55 eV (ref. 53), in the orthoferrites with moderate optical absorption ∼200 cm^−1^ at 1.55 eV (ref. 45) and in virtually transparent iron borate with absorption <100 cm^−1^ at 1.55 eV (ref. 54) shows that the optical modification of the *D*/*J* does not rely on laser heating due to optical absorption.

The maximum value of the oscillating magnetization in the samples is estimated to be ∼1 A m^−1^. Oscillations with such an amplitude can only be triggered if the laser excitation results in an ultrafast increase of the ratio *D*/*J* by >0.01% (see Supplementary Notes 4 and 5). Taking into account the parameters of our experiment, one can find that the sub-picosecond laser excitation with a fluence of ∼1 mJ cm^−2^ changes the potential energy of the magnetic system by ∼1 μJ cm^−2^ and acts as an effective magnetic field pulse of ∼0.01 T (see Supplementary Note 6). These values (normalized to the optical fluence) correspond to some of the largest effects of light on magnetic systems observed to date^19^,^22^.

To summarize, the demonstrated feasibility of a sub-picosecond modification of the fundamental exchange parameters *J* and *D* and the ratio between them opens wide prospects for optical control of magnetically ordered materials. The suggested mechanism is not restricted by any requirement on the crystal symmetry and must thus be applicable to other classes of magnetic materials. Given that in some materials isotropic magneto-refraction can be significantly larger than that in iron oxides, we foresee many opportunities to enhance the effects reported here. Finally, we anticipate that by tuning the wavelength of light, one should be able to affect selectively different exchange parameters in magnetic materials.

## Methods

### Samples

The crystals used in the present study were grown by floating zone melting (orthoferrites) and from the gas phase (iron borate and hematite) The orthoferrite samples were 60–100-μm thick and cut perpendicularly to the *z* axis (TmFeO_3_), the *x* axis (YFeO_3_, ErFeO_3_) and the *y* axis, (ErFeO_3_, DyFeO_3_). The iron borate FeBO_3_ sample (370-μm thick) and haematite α-Fe_2_O_3_ sample (500-μm thick) were cut perpendicularly to the *z* axis. The lateral size of all plates was ∼5 mm.

### Terahertz spectrometer

A conventional time-domain THz spectrometer was used in the measurements. The THz spectrometer was powered by a Ti:sapphire amplified laser, emitting a sequence of optical pulses (800 nm wavelength, 100 fs duration) with the repetition frequency of 1 kHz. Each laser pulse was divided into a stronger pump pulse and a weaker probe pulse. The pump spot size was larger than the aperture in the sample holder (∼2 mm in diameter) to provide a quasi-uniform excitation with a fluence of ∼1 mJ cm^−2^. The electric field of the emitted THz wave was measured by the electro-optical sampling technique. The sample was held inside a closed cycle, helium cryostat (15–300 K, 10^−4^ mbar).

### Theory of non-equilibrium exchange interactions

We use a general formalism in which magnetic interactions are obtained from a purely electronic model by introducing small time-dependent rotations of the spin quantization axes as was recently described in (ref. 48). For the general non-equilibrium case the evolution of the electronic model is described using the Schwinger–Keldysh/Kadanoff–Baym non-equilibrium action and partition function, with the effective action written in terms of Grassmann fields. By integrating over the electronic degrees of freedom in the rotated reference frame, an effective quadratic spin model is obtained in which the time-dependent exchange interaction parameters are identified from the mapping of the effective action to a time-dependent classical Heisenberg model. The resulting expressions turn out to be combinations of non-equilibrium electronic Green's functions and self-energies, which have to be evaluated numerically to assess the modification of exchange interaction by time-dependent perturbations of the electron model.

This method is implemented for the simplest model system that exhibits the physics of superexchange, which consists of a chain of three atoms, labelled as 0, 1 and 2. Atoms 0 and 2 correspond to transition metal sites with one partially filled *d* orbital and atom 1 contributes one filled (oxygen) *p* orbital. The Hamiltonian consists of a local part *H*_loc_ and a time-dependent hopping term *H*′(*t*):


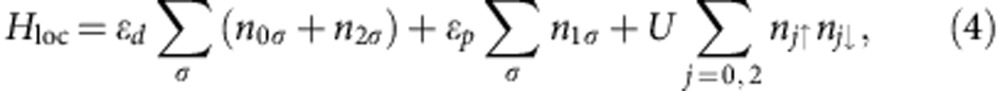



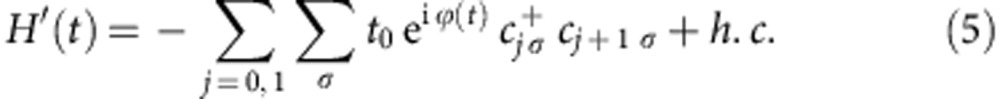


Here 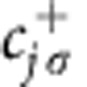 creates an electron with spin *σ*={↑,↓} at site *j*, and 
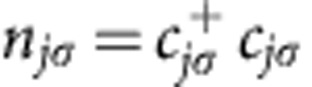
 is the number operator. The parameters *ɛ*_*d*_ and *ɛ*_*p*_ are the orbital energies of *d* and *p* orbitals, respectively, and *U* is the local (Hubbard) interaction energy associated with *d* orbitals. *H*′(*t*) accounts for hopping between *p* and *d* orbitals; *t*_0_ is the equilibrium hopping parameter, while *ϕ*(*t*) is the time-dependent Peierls phase, which absorbs the effect of the time-dependent electric field. In the Coulomb gauge and for a spatially uniform vector potential the Peierls phase is given by


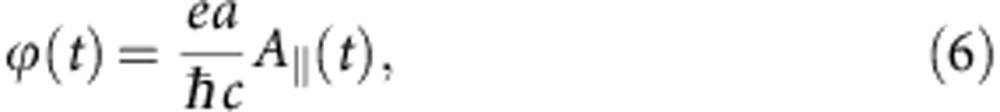


where *A*_||_(*t*) is the component of the vector potential parallel to the chain and *a* is the lattice spacing. In the chosen gauge, the electric field is related to the vector potential as 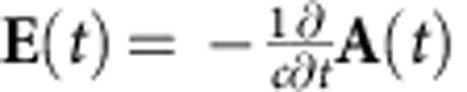. In the experimentally relevant regime the model is characterized by *U*, *U*+*ɛ*_*d*_−*ɛ*_*p*_>>*t*_0_ and a total filling of four electrons. To compute the non-equilibrium functions, this cluster model is solved numerically using exact diagonalization of the time-dependent Schrödinger equation. The electric field is taken as the product of an oscillatory component and a gradually changing envelope function with a rising time in the order of ∼10 periods of oscillation. The frequency *ω* of the oscillating part is below the charge-transfer gap, which prevents direct charge-transfer transitions, consistently with the experimental conditions.

Further details related to the theoretical and numerical methods are discussed in Supplementary Note 2.

## Additional information

**How to cite this article:** Mikhaylovskiy, R. V. *et al*. Ultrafast optical modification of exchange interactions in iron oxides. *Nat. Commun.* 6:8190 doi: 10.1038/ncomms9190 (2015).

## Supplementary Material

Supplementary Information Supplementary Figures 1-9, Supplementary Notes 1-6 and Supplementary References
