# Supplementary material for: Ultrafast optical modification of exchange interactions in iron oxides
Source: Nat Commun. 2015 Sep 16;6:8190. doi: 10.1038/ncomms9190 (PMC4595597; doi:10.1038/ncomms9190)
Supplement: Supplementary Information — Supplementary Figures 1-9, Supplementary Notes 1-6 and Supplementary References [file ncomms9190-s1.pdf]

Supplementary Figures

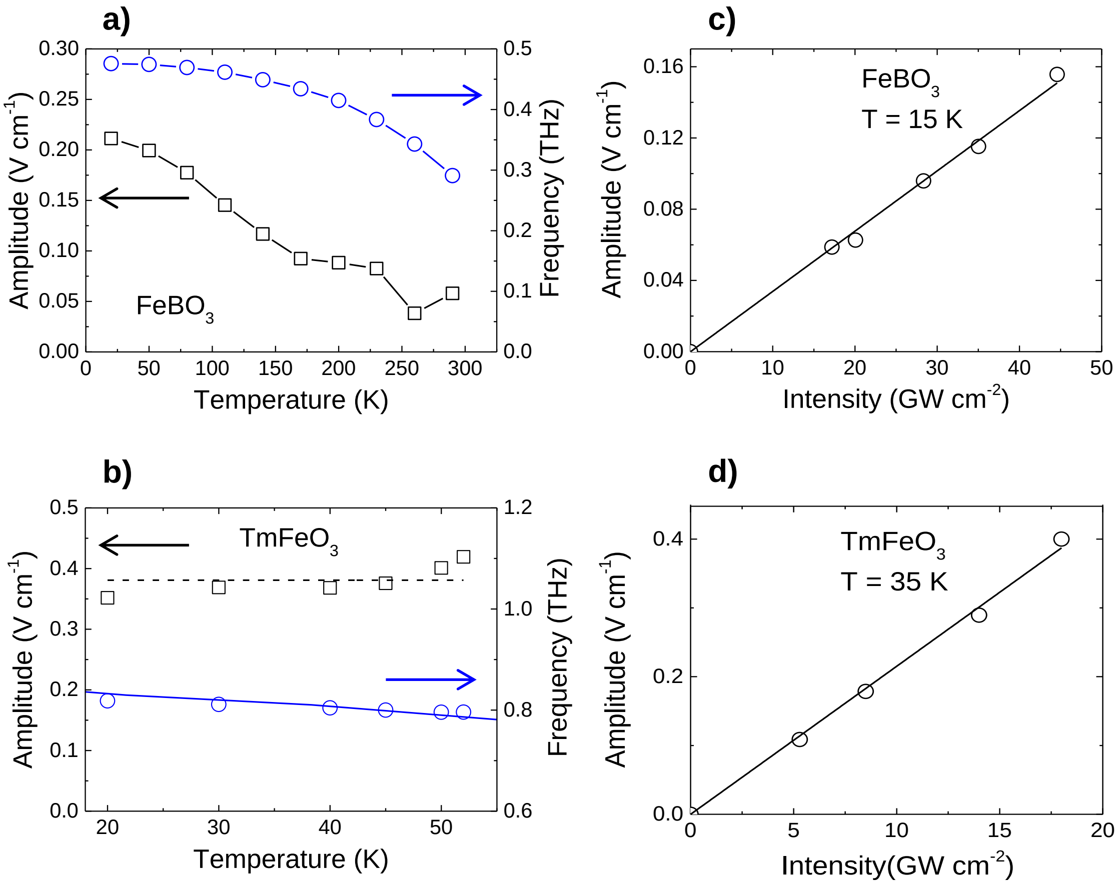

Supplementary Figure 1. Temperature and intensity dependence of the THz emission generated in $FeBO_3$ and $TmFeO_3$. a) The amplitude (open squares) and frequency (open circles) of the THz emission arising from the quasi-antiferromagnetic oscillations in the *z*-cut $FeBO_3$ are shown as a function of temperature. b) The amplitude (open squares) and frequency (open circles) of the THz emission arising from the quasi-antiferromagnetic oscillations in the *z*-cut $TmFeO_3$ vs temperature. The dashed line is a guide to the eye. The solid line shows the expected behavior of the quasi-antiferromagnetic resonance frequency extracted from Ref. 1. Note that the frequencies measured in our sample differ by ~ 10 % from those of Ref 1. c) The amplitude of the quasi-antiferromagnetic mode in $FeBO_3$ at 15 K is a linear function of the pump intensity. d) The amplitude of the quasi-antiferromagnetic mode in $TmFeO_3$ at 35 K scales linearly with the pump intensity.

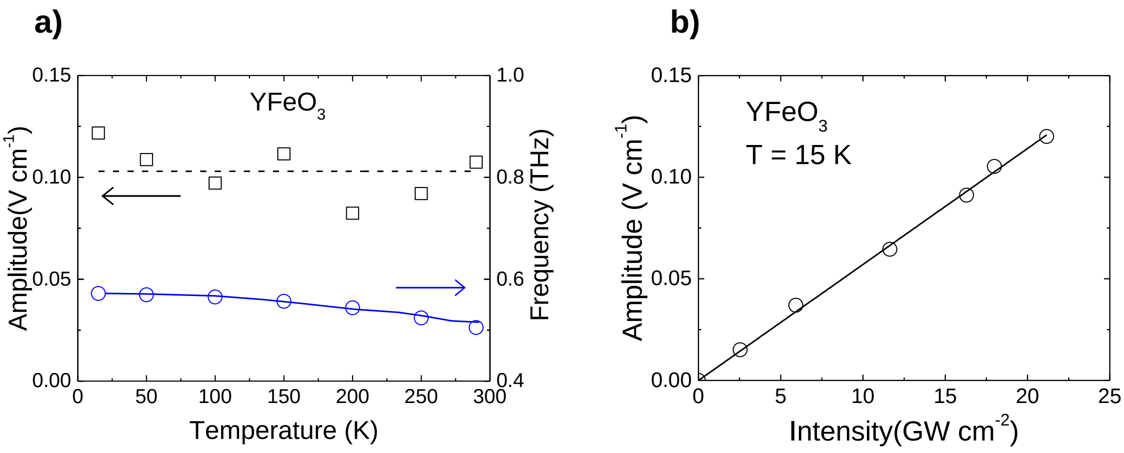

Supplementary Figure 2. Temperature and intensity dependence of the THz emission generated in $YFeO_3$ a) The amplitude (open squares) and frequency (open circles) of the THz emission from the *x*-cut $YFeO_3$ sample vs temperature is shown. The dashed line is a guide to the eye. The solid line shows the quasi-antiferromagnetic resonance frequency as a function of temperature taken from Ref. 2 with a correction shift of 60 GHz. b) The amplitude of the quasi-antiferromagnetic mode in $YFeO_3$ at 15 K is a linear function of the pump intensity.

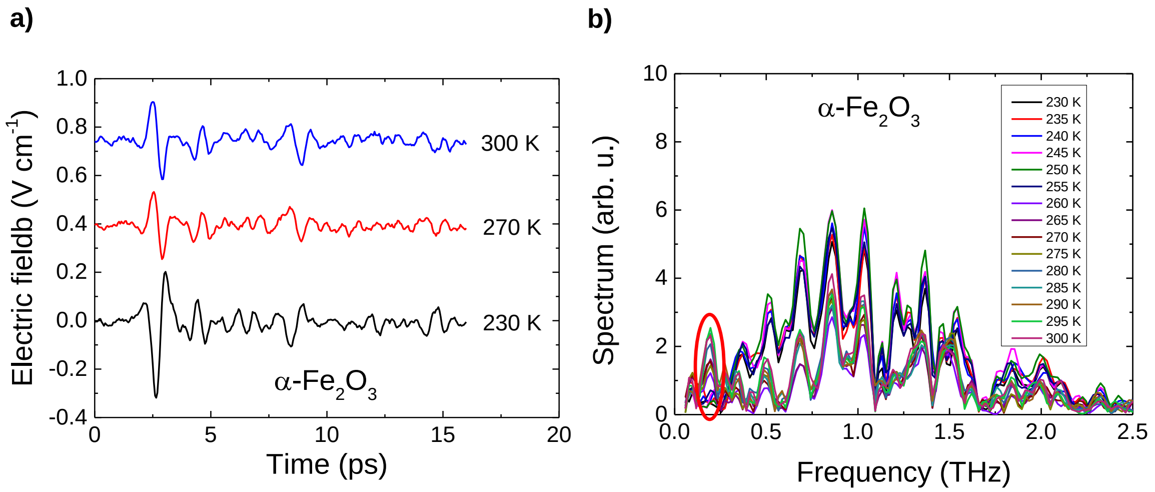

Supplementary Figure 3. THz emission generated in α-Fe_2O_3. a) Examples of waveforms generated in the *z*-cut α-Fe_2O_3 sample. b) The spectra of the emission. The peak centered at ~ 0.2 THz arising only above the Morin temperature is marked with a red oval.

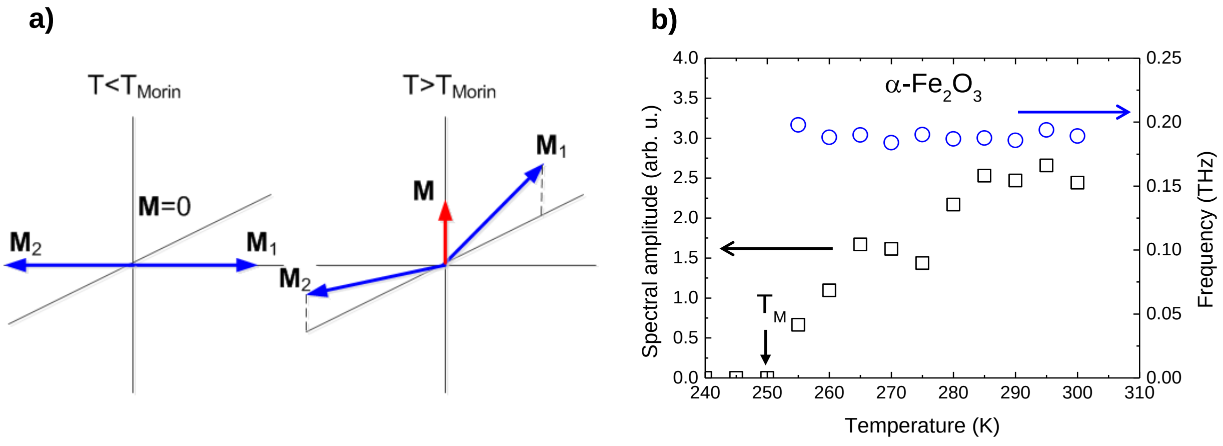

Supplementary Figure 4. a) The magnetic configuration of the hematite α-Fe_2O_3 below and above the Morin temperature. b) The amplitude (open squares) and frequency (open circles) of the THz emission component marked with red oval in Supplementary Fig. 4 (b) vs temperature. This component is attributed to the quasi-ferromagnetic mode which appears only in the canted antiferromagnetic phase above the Morin temperature T_M.

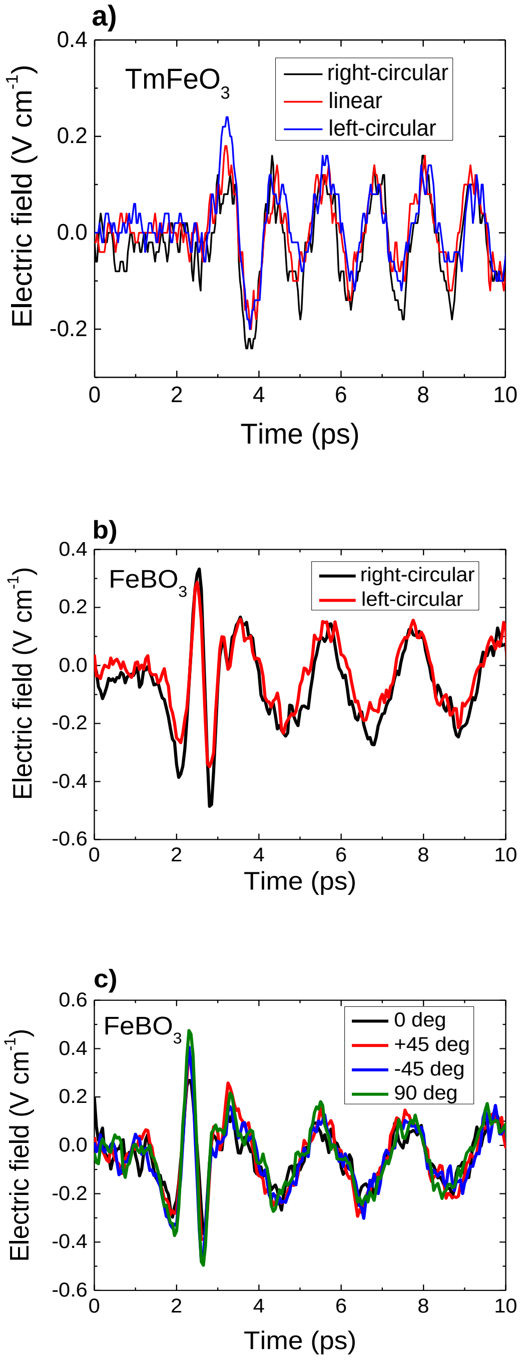

Supplementary Figure 5. THz emission generated in $FeBO_3$ and $TmFeO_3$ as a function of the pump polarization. a) The THz waveforms generated in the *z*-cut $TmFeO_3$ by optical pulses with linear or circular polarizations. b) The THz waveforms generated in the *z*-cut $FeBeO_3$ by circularly-polarized pump with opposite helicities. c) The THz waveforms generated in the *z*-cut $FeBeO_3$ by linearly-polarized pump. The angle in the legend of (c) corresponds to the orientation of the linear polarization with respect to the direction of the magnetization in the sample. The broadband pulse supposedly generated via electro-optical rectification arising from the surface nonlinearity is also seen at the start of the waveforms generated in $FeBO_3$. It indicates that the pump pulse arrival time equals ~ 1.5 ps.

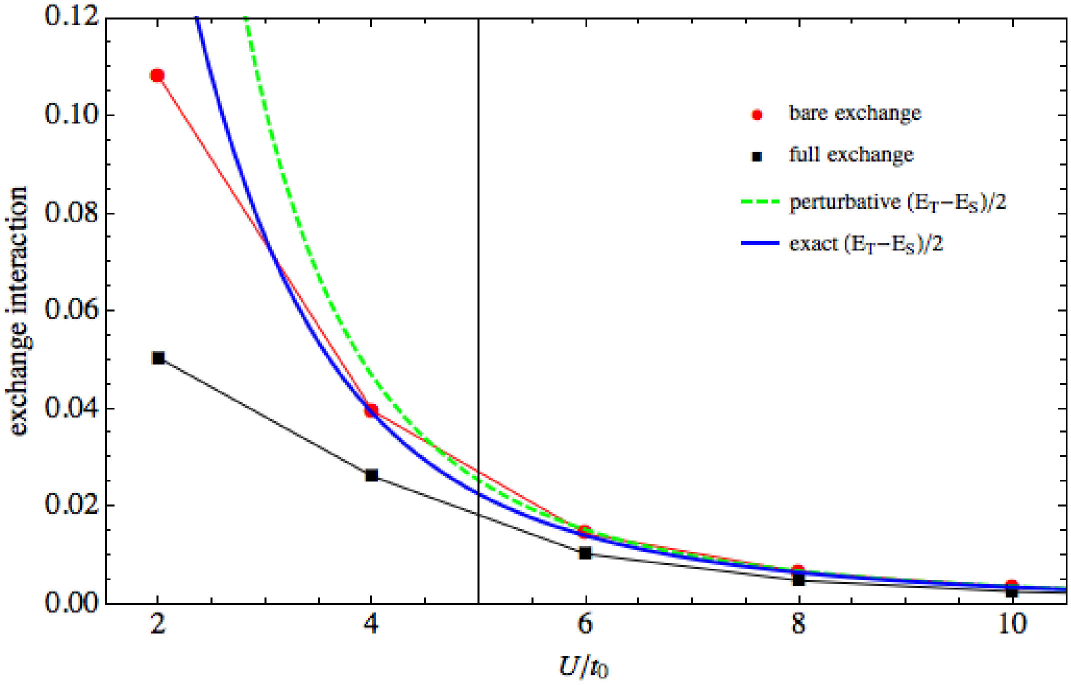

Supplementary Figure 6. Comparison of different expressions for the equilibrium exchange interaction as a function of U/t_0 .

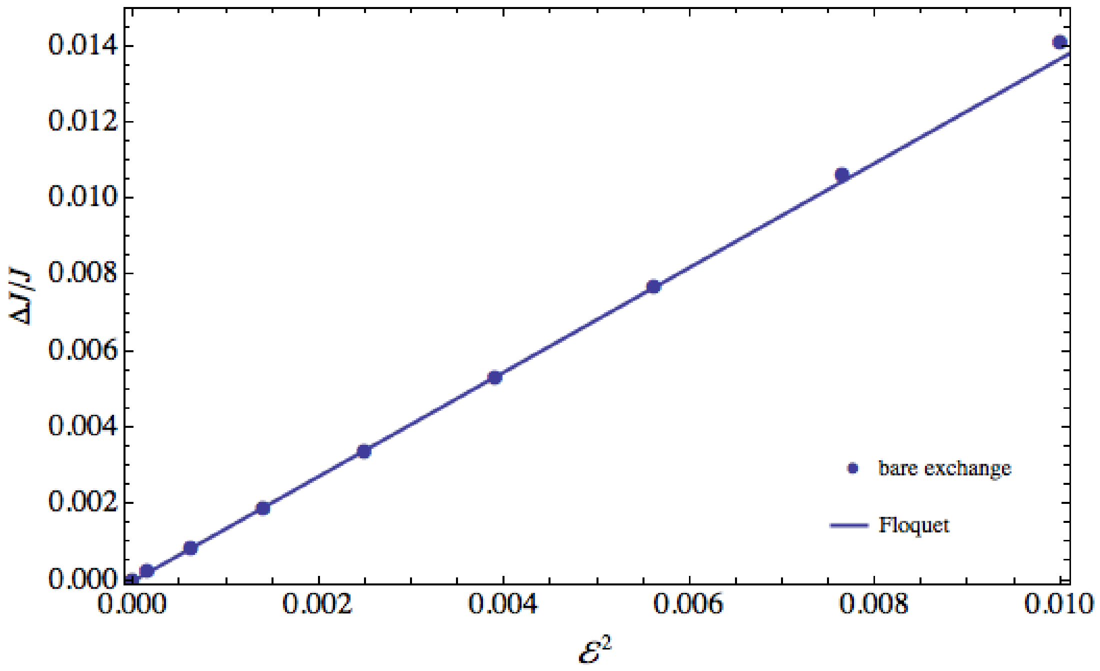

Supplementary Figure 7. Dependence of the exchange interaction on the applied electric field. The figure shows comparison of the amplitude of the modulation of the exchange calculated based on the general formulas (blue dots) and the analytical Floquet theory (solid line).

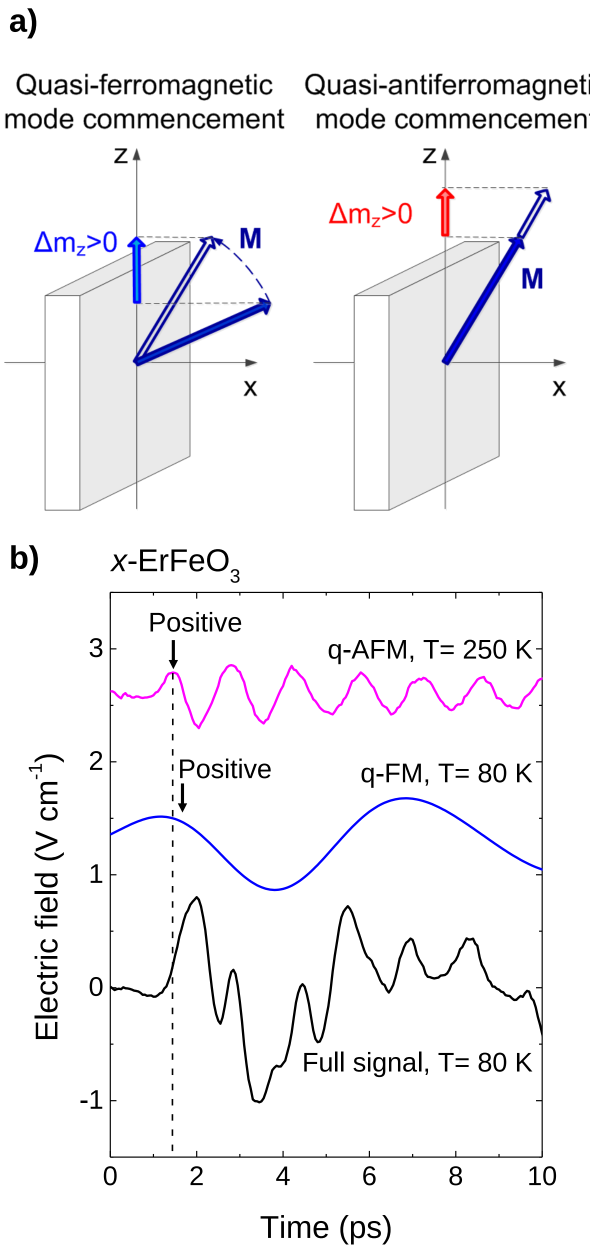

Supplementary Figure 8. Determination of the absolute sign of the change of *D*/*J* in the *x*-cut $ErFeO_3$. a) Due to the laser-induced reorientation the *z*-component of the magnetization must increase initially. At the same time the *z*-component of the magnetization increases due to the ultrafast change of the ratio *D*/*J*. b) The comparative analysis of the waveforms generated at the temperatures of the spin reorientation region and well above it demonstrates that the initial phases of the quasi-ferromagnetic (q-FM) and quasi-antiferromagnetic (q-AFM) modes have the same sign.

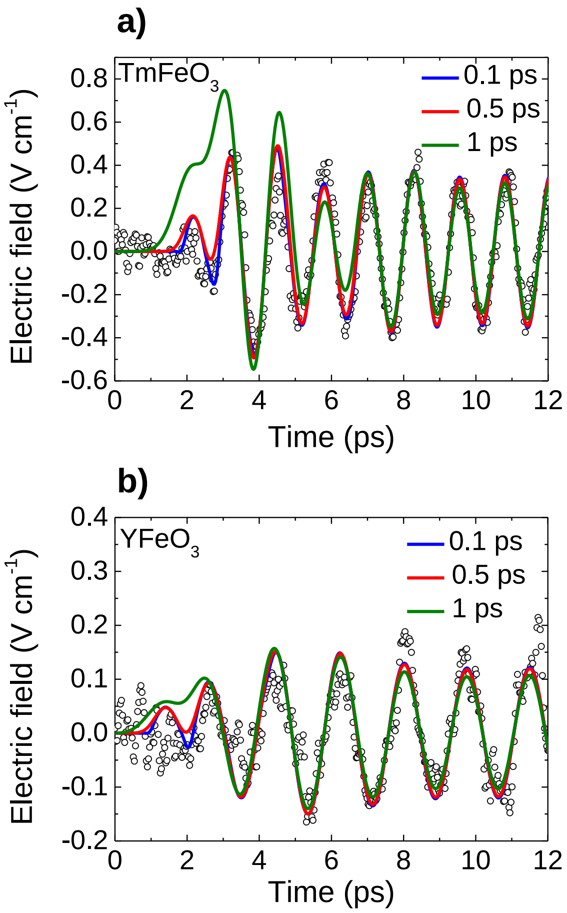

Supplementary Figure 9. Comparison of the experimental signals with the waveforms calculated theoretically. a) The signal generated in $TmFeO_3$ sample at 50 K (open circles) shown together with the theoretical waveforms (solid curves) calculated assuming different durations of the Gaussian torque b) The signal generated in $YFeO_3$ sample at 15 K (open circles) shown together with the waveforms calculated theoretically assuming different durations of the torque.

Supplementary Notes

Supplementary note 1 – Terahertz emission generated in the photo-excited α-Fe_2O_3

Supplementary Fig. 3 (a) shows the examples of the THz electric field generated in the *z*-cut α-Fe_2O_3 sample. It is seen from the Figure that the form of the signal is noticeably different from the quasimonochromatic signals generated in orthoferrites and iron borate. However, this is not surprising since the hematite crystal is highly absorbing at 800 nm, in contrast to the other materials measured in our experiments. The signal is dominated by the broadband THz pulse and its multiple Fabry-Perot replicas. This broadband pulse arises from either surface nonlinearity or magnetic dipole-electric quadrupole nonlinearity although investigation of its exact origin is beyond the scope of the present study. However, one can observe [see Supplementary Fig. 3 (b)] that above 250 K a spectral component at ~0.2 THz arises that is not present in the spectrum of the THz transient. The frequency of this component matches perfectly the frequency of the quasi-antiferromagnetic resonance in the hematite [3]. Moreover, this spectral component does not depend on the pump polarization, while the rest of the emission spectrum does depend on the polarization of the pump,l which indicates their different origins. Importantly, the α-hematite exhibits a first-order phase transition from a purely antiferromagnetic state to the canted state at the critical Morin temperature $T_M = 250$ K. Thus, only in the canted state above T_M one anticipates the excitation of the quasi-antiferromagnetic resonance due to the modification of the exchange parameters. Indeed, the quasi-antiferromagnetic mode is observed only above the Morin point (Supplementary Fig. 4).

Supplementary note 2 – Microscopic theory of non-equilibrium exchange

To demonstrate theoretically the feasibility of the modification of the super-exchange interaction, we adapt a quantum theory [4] that was recently developed for describing non-equilibrium magnetic interactions in strongly correlated systems. We specialize the application of this framework to a simple cluster model that mimics the experimental system (in particular, α-Fe_2O_3) and we solve this model numerically. Furthermore, to provide additional theoretical understanding, we derive analytical results from Floquet theory for the same cluster model. Both the numerical and analytical results demonstrate an enhancement of the exchange interaction that scales linearly with the intensity of the electric field.

Quantum theory of non-equilibrium magnetic interactions

Our theory exploits the non-equilibrium Green-function formalism developed by Schwinger [5], Keldysh [6], Kadanoff and Baym [7]. The electronic partition function Z is written as a path integral over fermionic (Grassmann) fields of the exponential of a non-equilibrium action $S[\bar{\phi},\phi]$, i.e.,

$$Z = \int \mathrm{D}\left[\bar{\phi},\phi\right] \exp\left(\mathrm{i}\, S\left[\bar{\phi},\phi\right]\right). \tag{1}$$

The effective action describes the system in equilibrium for $t < 0$, going out of equilibrium for $t > 0$. Fermion fields $\phi_{a\sigma}$ are labelled by indices a and $\sigma = \{\uparrow, \downarrow\} = \{+, -\}$, referring respectively to site (we are considering the single-band case below) and spin single-particle states. The spin quantization axis of the ϕ fermions is along the unit vector $\mathbf{u}_z$. To study the spin excitations on top of the equilibrium ground state, we apply time- and site- dependent rotations to the local spinors, defined as $\phi_a = \left(\phi_{a\uparrow}, \phi_{a\downarrow}\right)^T$, in order to transform the old fermion fields $\phi_{a\sigma}$ into new fields $\psi_{a\sigma}$ having their spins aligned with time-dependent unit vectors $\sigma \mathbf{e}_a(t)$, which are interpreted as the directions of (classical) magnetic moments. The deviations of the $\mathbf{e}_a(t)$'s from the equilibrium direction $\pm\mathbf{u}_z$ are described by auxiliary (Holstein-Primakoff) boson fields; for low-energy excitations we assume such deviations to be small, obtaining an action quadratic in the bosons (this corresponds to small mixing between quantum states with different total spin). By integrating out the fermion fields ψ an effective bosonic action is obtained and we finally map the bosons to the $\mathbf{e}_a(t)$ fields to explicitly identify the

spin-spin interactions. The coefficients describing the magnetic interactions are expressed in terms of non-equilibrium electronic Green's functions and related self-energies, which generalize the equilibrium formalism [8] to include the effects of an external time-dependent field.

Assuming that the spin dynamics is slow with respect to electronic hopping processes, we obtain the following formula for the non-equilibrium exchange coupling between sites *a* and *b*:

$$\eta_{ab}\, j_{ab}(t) = \frac{1}{4}\mathrm{Re}\left[F_{ab}(t)\right] + \frac{1}{4}\int_0^t dt'\ \mathrm{Im}\left[A_{ab}^{>}(t',t) - A_{ab}^{<}(t',t)\right], \tag{2}$$

where $\eta_{ab} = +1\,(-1)$ if the ground-state spin correlation function of sites *a* and *b* is antiferromagnetic (ferromagnetic), and the quantities to be computed are the following (see Eqs. (107) and (124) in [4]):

$$\begin{aligned} A_{ab}^{>}(t,t') = \sum_c \sum_d & \left[-\delta_{ac}\,\mathrm{i}\frac{\overrightarrow{\partial}}{\partial t} + \left(1 - \overrightarrow{P}_{ac}\right) t_{ac}(t) \right] G_{cb}^{<\downarrow}(t,t')\ G_{da}^{>\uparrow}(t',t) \\ & \times \left[-\delta_{bd}\ \mathrm{i}\frac{\overleftarrow{\partial}}{\partial t'} + t_{bd}(t')\left(1 - \overleftarrow{P}_{bd}\right) \right]. \end{aligned} \tag{3}$$

$A_{ab}^{<}(t,t')$ is obtained from this equation by exchanging > and < in the right-hand side, and for $a \neq b$,

$$F_{ab}(t)\big|_{a\neq b} = \mathrm{i}\,\overline{\Sigma}_{ab}^{\downarrow}(t)\, G_{ba}^{<\uparrow}(t,t) + \mathrm{i}\,G_{ab}^{<\downarrow}(t,t)\,\overline{\Sigma}_{ba}^{\uparrow}(t). \tag{4}$$

The above quantities are expressed in terms of the non-equilibrium Green’s functions,

$$G_{ba}^{<\sigma}(t,t') = \mathrm{i}\left\langle \hat{\psi}_a^{+\sigma}(t')\ \hat{\psi}_b^{\sigma}(t)\right\rangle, \quad G_{ba}^{>\sigma}(t,t') = -\mathrm{i}\left\langle \hat{\psi}_b^{\sigma}(t)\,\hat{\psi}_a^{+\sigma}(t')\right\rangle, \tag{5}$$

and in terms of the time-dependent Hartree-Fock component of the self-energy $\overline{\Sigma}(t)$. Furthermore, in Eq. (3) the arrows above the operators define the directions along which the operators act, P_{ab} is the operator interchanging indices *a* and *b* in the functions it acts upon, and $t_{ab}(t)$ are hopping matrix elements, which depend on time in the presence of a time-dependent external field.

For the numerical calculations, it is convenient to work out the derivatives analytically, which yields the lesser and greater components of [1]

$$A_{ab}(t,t') = R_{ab}^{\downarrow}(t,t')\,R_{ba}^{\uparrow}(t',t) + S_{ab}^{\downarrow}(t,t')\,S_{ba}^{\uparrow}(t',t) - G_{ab}^{\downarrow}(t,t')\,T_{ba}^{\uparrow}(t',t) \\ - T_{ab}^{\downarrow}(t,t')\,G_{ba}^{\uparrow}(t',t)\,. \tag{6}$$

Here we have introduced the quantities

$$R_{ab}^{\sigma}(t,t') = [\Sigma * G]_{ab}^{\sigma}(t,t'), \tag{7a}$$

$$S_{ab}^{\sigma}(t,t') = [G * \Sigma]_{ab}^{\sigma}(t,t'), \tag{7b}$$

$$T_{ab}^{\sigma}(t,t') = \Sigma_{ab}^{\sigma}(t,t') + [\Sigma * G * \Sigma]_{ab}^{\sigma}(t,t'), \tag{7c}$$

where $\Sigma_{ab}^{\sigma}(t,t')$ is the self-energy, which accounts for the electron correlations.

It must be noticed that the effective exchange parameter in Eq. (2) is not the “bare” exchange interaction between the quantum spins in sites *a* and *b*, since it also incorporates a term which describes the variation with time of the magnitudes of the local magnetic moments (non-Heisenberg effects). The derivation leading to Eq. (2), in fact, allows to map an electronic system onto an effective system of time-dependent interacting unit vectors, which can be interpreted as the directions of classical local spins (see Ref.[8]). While these directions appear as dynamical variables in our formulation, the magnitudes of the respective quantum spins, as well as possible other quantum effects, are included in the definition of the parameters of Eq. (2), which measure the strengths of the interactions between the unit vectors. In the absence of symmetry breaking

---

[1] The symbols $A^{>}$ and $A^{<}$ in Eq.(3) are defined according to the conventions used in Ref.[4]; in particular, time variables t and t' are defined on the real axis. In Eq.(6) time variables are defined on the Keldysh contour. The correspondence between Eqs.(3) and (6) is given by $A^{>}(t,t') = A(t_{+},t'_{-})$ and $A^{<}(t,t') = A(t_{-},t'_{+})$, where ± indicate times on the upper and lower branches of the Keldysh contour, respectively. Note that this is different from the conventional definition of lesser and greater components of Keldysh functions.

(which is the case that we consider here), we obtain the bare exchange parameters (i.e., the strengths of the interacions between the *spins*) as

$$J_{ab}(t) = \frac{j_{ab}(t)}{\langle \mathbf{s}_a(t) \cdot \mathbf{s}_b(t) \rangle}, \tag{8}$$

where $\langle \mathbf{s}_a(t) \cdot \mathbf{s}_b(t) \rangle$ is the equal-times spin-spin correlation function.

Minimal model for super exchange

As minimal model for super-exchange we consider a chain of three atoms denoted 0, 1 and 2 [9, 10]. Atoms 0 and 2 correspond to transition metal sites with one partially filled *d*-orbital and atom 1 contributes one filled (oxygen) *p*-orbital. The Hamiltonian consists of a local part H_{loc} and a time-dependent hopping term $H'(t)$:

$$H_{\mathrm{loc}} = \varepsilon_d \sum_{\sigma} (n_{0\sigma} + n_{2\sigma}) + \varepsilon_p \sum_{\sigma} n_{1\sigma} + U \sum_{j=0,2} n_{j\uparrow} n_{j\downarrow}, \tag{9a}$$

$$H'(t) = -\sum_{j=0,1} \sum_{\sigma} t_0 \, \mathrm{e}^{\mathrm{i}\varphi(t)} c^{+}_{j\sigma} c_{j+1\,\sigma} + h.c. \quad . \tag{9b}$$

Here $c^{+}_{j\sigma}$ creates an electron with spin $\sigma = \{\uparrow, \downarrow\}$ at site *j*, and $n_{j\sigma} = c^{+}_{j\sigma} c_{j\sigma}$ is the number operator. We choose the zero of the energy as $\varepsilon_d + \varepsilon_p = 0$, and define $\Delta = \varepsilon_d - \varepsilon_p$; *U* is the local (Hubbard) interaction energy associated with *d*-orbitals, that is, $U = \iint d\vec{r}\, d\vec{r}' \left|\psi_{j,d}(\vec{r})\right|^2 V(\vec{r} - \vec{r}') \left|\psi_{j,d}(\vec{r}')\right|^2$, where $\psi_{j,d}(\vec{r})$ is the wave function of the *d*-orbital localized on site *j* and $V(\vec{r} - \vec{r}')$ is the effective Coulomb interaction energy between electrons at positions $\vec{r}$ and $\vec{r}'$. The single-electron Hamiltonian $H'(t)$ accounts for time-dependent hoppings between *p*- and *d*-orbitals. Specifically, the equilibrium hopping parameter

$t_0 = -\int d\vec{r}\; \psi^{*}_{0,d}(\vec{r})\, H_1(\vec{r})\, \psi_{1,p}(\vec{r})$, with the dimensions of energy, is the matrix element between the *d*- and *p*-orbitals localized on iron and on oxygen ions, respectively of the equilibrium single-electron Hamiltonian $H_1(\vec{r})$, which includes the kinetic energy of the electrons, the interaction with the ions, as well as any other single-electron time-independent potential.

The time-dependent electric field $E(t)$ is included in $H'(t)$ (see Eq. 9b) by means of the time-dependent Peierls substitution [11- 13], which is equivalent to multiplying the equilibrium hopping by a time-dependent phase factor. In the Coulomb gauge (zero scalar potential) and for a spatially uniform vector potential the Peierls phase becomes

$$\varphi(t) = \frac{ea}{\hbar c} A_{\parallel}(t), \tag{10}$$

where $A_{\parallel}(t)$ is the component of the vector potential parallel to the chain and a is the lattice spacing. The electric field is then related to the vector potential as $\mathbf{E}(t) = -\frac{1}{c}\frac{\partial}{\partial t}\mathbf{A}(t)$. For periodic driving field $E_{\parallel}(t) = E_0 \cos(\omega t)$ along the chain we obtain

$$\varphi(t) = -\varepsilon \sin(\omega t), \tag{11}$$

where the amplitude is given in terms of the dimensionless parameter

$$\varepsilon = \frac{eaE_0}{\hbar\omega}. \tag{11}$$

For the numerical solution of the periodically driven cluster model we slowly switch on the Peierls phase using an error function envelope

$$\varphi(t) = -\frac{1}{2}\varepsilon \sin(\omega t)\left[\mathrm{erf}\left(\alpha\left(t - t_1\right)\right) + 1\right]. \tag{12}$$

where (for a given ω) the parameters α and t_1 are chosen such that $\varphi(0) = 0$ and the rise time takes about 10 oscillation periods.

Numerical computation of non-equilibrium exchange parameters

A numerically exact solution of the 3-site super-exchange model out of equilibrium is obtained by solving the time-dependent Schrödinger equation using exact diagonalization. From the time evolution of the states we evaluate the following correlation functions using the Lehmann representation:

$$G^{\sigma}_{ab}(t,t') = -\mathrm{i}\left\langle \hat{T}_C\, c_{a\sigma}(t)\, c^{+}_{b\sigma}(t') \right\rangle, \tag{13a}$$

$$R_{ab}^{\sigma}(t,t') = -\mathrm{i}\left\langle \hat{T}_C\; n_{a\bar{\sigma}}(t)\, c_{a\sigma}(t)\, c_{b\sigma}^{+}(t') \right\rangle U_a, \tag{13b}$$

$$S_{ab}^{\sigma}(t,t') = -\mathrm{i}\left\langle \hat{T}_C\; c_{a\sigma}(t)\, c_{b\sigma}^{+}(t')\, n_{b\bar{\sigma}}(t') \right\rangle U_a, \tag{13c}$$

$$T_{ab}^{\sigma}(t,t') = -\mathrm{i}\left\langle \hat{T}_C\; n_{a\bar{\sigma}}(t)\, c_{a\sigma}(t)\, c_{b\sigma}^{+}(t')\, n_{b\bar{\sigma}}(t') \right\rangle U_a\, U_b, \tag{13d}$$

where $\hat{T}_C$ is the time-ordering operator along the Keldysh contour and U_a is the Coulomb interaction at site *a*. By computing products like $R_{ab}^{\downarrow}(t,t')R_{ba}^{\uparrow}(t,t')$ and by combining all terms we obtain the quantity $A_{ab}(t,t')$.

In addition, we evaluate the spin-spin correlation function as

$$\langle \mathbf{s}_a(t)\cdot \mathbf{s}_b(t)\rangle = \sum_{\sigma}\left[\frac{1}{2}\left\langle c_{a\sigma}^{+}(t)\, c_{a\bar{\sigma}}(t) c_{b\bar{\sigma}}^{+}(t)\; c_{b\sigma}(t)\right\rangle + \frac{1}{4}\left\langle n_{a\sigma}(t)\, n_{b\sigma}(t) - n_{a\sigma}(t)\, n_{b\bar{\sigma}}(t)\right\rangle\right]. \tag{14}$$

For the cluster model we consider a filling with 4 electrons and total spin $S_z = 0$, and prepare the system at $t = 0$ in the ground state at low temperature.

In equilibrium the exchange interactions are extracted at zero electric field by evolving the system along the real time axis to extract $A_{ab}(t,t') = A_{ab}(t-t')$ for $a, b = 0, 2$. The full (static) exchange interaction j_{ab}^{0} is obtained from Eq.(2), by taking $t = t_{*}$ large enough,

$$j_{ab}^{0} = j_{ab}(t_{*}). \tag{15}$$

We use a Gaussian window of length L to ensure a smooth cutoff of the upper integration boundary. Numerically converged results, independent of L and t_{*}, are obtained for $t_{*} > L > j_{ab}^{-1}$. The bare exchange interaction, Eq. (8) is then computed as

$$J_{ab}^{0} = \frac{j_{ab}^{0}}{\langle \mathbf{s}_a(0)\cdot \mathbf{s}_b(0)\rangle}. \tag{16}$$

Out of equilibrium, the hopping matrix elements are modulated by the Peierls phase, and the $j_{ab}(t)$ and $\langle \mathbf{s}_a(t)\cdot \mathbf{s}_b(t)\rangle$ oscillate with the electric field. In this case, we compute the effective exchange interaction by averaging the bare exchange interaction over the period T of the field

$$J_{ab}^{\varepsilon} = \frac{1}{T}\int_{t_*}^{t_*+T} dt \left[\frac{j_{ab}(t)}{\langle \mathbf{s}_a(t)\cdot \mathbf{s}_b(t)\rangle} \right]. \tag{17}$$

The result is found to be independent on t_* in the quasi-equilibrium state formed after slowly ramping up the Peierls phase.

Analytical Floquet theory

To gain additional understanding on the electrical control of the exchange interaction we use an alternative approach based on Floquet theory. For small amplitudes ε, relevant to the experimental conditions, this allows us to derive analytical formulae for the non-equilibrium exchange interaction under periodic driving of the 3-site cluster model.

To discuss the superexchange mechanism in the unperturbed system (ε = 0), we consider a filling of the cluster with 4 electrons, and apply perturbation theory to describe the low-energy states. For large Coulomb interaction (U, $U + \Delta >> t_0$) the low-energy sector of the Hilbert space includes the states with two electrons occupying the oxygen p-orbital and one electron in each of the two transition metal d-orbitals $H_0 = \{ |\downarrow, 2, \uparrow\rangle, |\uparrow, 2, \downarrow\rangle, |\downarrow, 2, \downarrow\rangle, |\uparrow, 2, \uparrow\rangle \}$. The symbols in $|\quad\rangle$ denote the occupation of the orbitals 0, 1, 2, from left to right, which can be either empty (0), singly occupied $(\uparrow, \downarrow)$, or doubly occupied (2). Virtual charge-transfer excitations due to the hopping t_0 to states $|2, 2, 0\rangle, |0, 2, 2\rangle$ etc. lead to a shift of the levels. A splitting $E_T - E_S$ of singlet $\left(|\downarrow, 2, \uparrow\rangle - |\uparrow, 2, \downarrow\rangle \right)/\sqrt{2}$ and triplet levels occurs in fourth order,

$$E_T - E_S = 4t_0^4 \left(\frac{1}{U_1^2 U} - \frac{1}{U_1^3} \right). \tag{18}$$

where $U_1 = U + \Delta$. The low-energy Hamiltonian is thus an antiferromagnetic Heisenberg model with exchange interaction $J = (E_T - E_S)/2$.

For a periodically driven system, solutions of the time-dependent Schrödinger equation can be found in terms of the Floquet modes of the form

$$|\psi(t)\rangle = e^{-i\zeta_\alpha t} |\psi_\alpha(t)\rangle, \tag{19}$$

where $|\psi_\alpha(t)\rangle = |\psi_\alpha(t+T)\rangle$ is periodic with $T = 2\pi/\omega$ [14-15]. To determine $|\psi_\alpha(t)\rangle$ and the Floquet spectrum ζ_α, we expand $|\psi_\alpha(t)\rangle$ as

$$|\psi_\alpha(t)\rangle = \sum_n \mathrm{e}^{-\mathrm{i}\omega n t} |\psi_{\alpha,n}(t)\rangle . \tag{20}$$

The Schrödinger equation $\mathrm{i}\hbar\, \partial_t |\psi(t)\rangle = H(t)\, |\psi(t)\rangle$ gives

$$(H_{\mathrm{loc}} + n\hbar\omega)|\psi_{\alpha,n}(t)\rangle + \sum_m H'_m |\psi_{\alpha,n+m}(t)\rangle = \zeta_\alpha |\psi_{\alpha,n}(t)\rangle , \tag{21}$$

where H'_n are the Fourier components of the hopping term $H'(t)$,

$$H'_n = \frac{1}{T}\int_0^T dt\, H'(t)\mathrm{e}^{\mathrm{i}\omega n t} . \tag{22}$$

The quasi-energies parametrically evolve with ε from the unperturbed energies $\zeta_\alpha^0 = E_\alpha(+n\hbar\omega)$ to the perturbed levels $\zeta_\alpha(\varepsilon)$. As a function of time, the amplitude evolves with the pulse envelope, and thus has typically only small variation on the timescale of the period T. Under these conditions, the system would adiabatically evolve from the low energy states of $H(\varepsilon = 0)$ into the corresponding Floquet modes, and the slow dynamics during the pulse (in particular, precessional motion of spins) is governed by an effective Hamiltonian that is determined by the level spectrum $\zeta_\alpha(\varepsilon)$. In particular, we can obtain the non-equilibrium exchange interaction from the singlet-triplet splitting as $J(\varepsilon) = (\zeta_{\mathrm{T}}(\varepsilon) - \zeta_{\mathrm{S}}(\varepsilon))/2$.

The experiment is performed in the regime of weak perturbation $\varepsilon << 1$ (from the estimates in the main text we get ε ~0.04). In this case, the level splitting can be determined analytically by standard degenerate perturbation theory for the extended eigenvalue problem, where we keep all terms up to fourth order in t_0 and second order in ε. For the Fourier transform of the hopping term we find

$$H'_n = -t_0\, J_n(\varepsilon) \sum_\sigma \left(c^+_{2\sigma} c_{1\sigma} + c^+_{1\sigma} c_{0\sigma} \right) + (-1)^n \times h.c., \tag{23}$$

where $J_n(x) = \frac{1}{2\pi}\int_{-\pi}^{\pi} ds\, \mathrm{e}^{\mathrm{i}[x\sin(s) - ns]}$ is the n-th order Bessel function. The term $n = 0$ thus describes a reduction of the hopping with the factor $J_0(\varepsilon) = 1 - \varepsilon^2/4 + O(\varepsilon^2)$. At the extremely large

amplitude $\varepsilon \approx 2.405$, the effective hopping vanishes which is known as coherent destruction of tunneling [16-17]. The terms H'_n with $n \neq 0$ couple to higher Floquet sectors, which corresponds to a dressing of the levels with virtual absorption/emission of *n* photons. Because $J_n(\varepsilon) \sim \varepsilon^n$ for $\varepsilon \to 0$, we can restrict ourselves to $n = \pm 1$ ($H'_{n \neq 0}$ does not contribute in first order perturbation theory). Summing up all hopping processes in $H'_{\pm 1}$ and H'_0 of the perturbation theory yields the result of the main text,

$$\Delta J = \frac{\varepsilon^2 t_0^4}{2}\left(\sum\nolimits_{\pm} \left[\frac{1}{U_1 \pm \hbar\omega} + \frac{1}{U_1} \right]^2 \frac{1}{U \pm \hbar\omega} - \frac{4}{U_1^2 U} - \frac{4}{U_1^3} \right). \tag{24}$$

Exchange can be strengthened for low frequencies due to the effective lowering of the charge transfer energy by a virtual photon, and weakened for very high frequencies, where the coupling to higher Floquet bands is irrelevant and the coherent destruction of tunneling dominates. In experiments we typically have $\hbar\omega \sim U_1/2$, hence we anticipate a strengthening of the exchange interaction by the photon-assisted charge-transfer excitations under experimental conditions. We also note that in the strict limit $\omega \to 0$ (which is not relevant for experiments since it requires very long laser pulses) a small negative change ΔJ appears when $\Delta > 0$.

Let us remark that Floquet theory makes predictions that will not be further discussed in the current manuscript. For example, the expression Eq. (24) shows that the effect of the electric field on the exchange interaction is strongly enhanced close to the resonance (where in a solid, however, one has strong absorption). In addition, different effects may occur in the non-perturbative regime, where the coupling to Floquet higher sectors is not negligible. Finally, the current model is clearly just a minimal model for laser-controlled super-exchange. A much richer behavior can be expected if more orbitals are included in the description.

Results

Before analyzing the effect of an electric field out of equilibrium, we study in equilibrium the quality of the general formulas Eq. (8) and Eq. (16), as well as the perturbative analytical expression Eq. (18) against the exact singlet-triplet splitting of the 3-site cluster. The result is shown in Supplementary Fig. 6, where the different calculations of the exchange interaction are plotted as functions of U/t_0 for $\Delta/t_0 = 0.5$ and low temperature $\beta t_0 = 8000$, $\beta = 1/k_B T$.

It is observed that the full exchange interaction (j_{02}^{0}, black squares) introduces a systematic underestimation compared to the exact singlet-triplet splitting (blue solid lines) for all values of U. Instead, the bare exchange interaction (J_{02}^{0}, red discs) shows excellent numerical agreement already for relatively small $U/t_0 = 4$. We attribute the deviations at smaller U to the neglect of vertex corrections in the general formulas. The perturbative analytical expression Eq. (24) (green dashed line) is quite accurate already at $U/t_0 = 6$.

Supplementary Fig. 7 shows the relative change of the exchange interaction $\Delta J/J$ in the periodically driven cluster model as a function of ε^2. For the numerical evaluation of the general formulas we slowly switched on the electric field using a rise time of 10 oscillation periods of the electric field pulse, using the model parameters $U/t_0 = 6$, $\Delta/t_0 = 0.5$, $\hbar\omega/t_0 = 3$, $\beta\, t_0 = 2000$. The results show an excellent quantitative agreement between the numerical results of the general formula, J_{ab}^{ε} from Eq. (17), and the analytical Floquet theory Eq.(24), demonstrating an enhancement of the exchange interaction that scales linearly with the intensity of the electric field. The order of magnitude of the effect in absolute numbers is discussed in the main text.

The relation between the modification of the exchange interaction in a Fe-O-Fe cluster and of the macroscopic exchange in the crystal

Our model computes the modification of the exchange interaction along one Fe-O-Fe bond. Hence the model predicts that bonds with a different projection of the electric field experience a different modification of exchange. However, as long as the effect is proportional to the intensity of the electric field, this still yields an isotropic effect in the interaction Hamiltonian of Eq. (1) of the main text, provided that the crystal symmetry allows this.

To understand the isotropic nature of Eq. (1), it is instructive to consider a cubic lattice with bonds along the crystal axes. Assuming that the exchange interaction J_{ij} is modified by ΔJ_{ij}, the resulting modification of the exchange energy can be written as $\sum_{\text{all bonds}} \Delta J_{ij} \mathbf{S}_i \mathbf{S}_j = \sum_{\text{bonds}\,x} \Delta J_{xij} \mathbf{S}_i \mathbf{S}_j + \sum_{\text{bonds}\,y} \Delta J_{yij} \mathbf{S}_i \mathbf{S}_j + \sum_{\text{bonds}\,z} \Delta J_{zij} \mathbf{S}_i \mathbf{S}_j$. Using the cubic symmetry we can write $\Delta J_{xij} = Y_{ij} |E_x|^2$, $\Delta J_{yij} = Y_{ij} |E_y|^2$, and $\Delta J_{zij} = Y_{ij} |E_z|^2$, where the coefficient Y_{ij} is the same for all three terms. Hence we have

$\sum_{\text{all bonds}} \Delta J_{ij} \mathbf{S}_i \mathbf{S}_j = \sum_{\text{all bonds}} Y_{ij} \mathbf{S}_i \mathbf{S}_j \left(|E_x|^2 + |E_y|^2 + |E_z|^2 \right) = \sum_{\text{all bonds}} Y_{ij} \mathbf{S}_i \mathbf{S}_j |\mathbf{E}|^2$ in agreement with the isotropic Hamiltonian of Eq. (1) of the main text.

In a more general case, the symmetry of the (inverse) magneto-optical effect is determined by the crystal structure, which imposes similar symmetry requirements for both the macroscopic theory (Eq. 1 of the main text) and more elaborate microscopic theory of an extended crystal. For example, since the model predicts that the strength is proportional to the dimensionless amplitude $\varepsilon = eaE_0/\hbar\omega$, with a being the bond length, we anticipate slight deviation from full isotropy when the system is not perfectly cubic (e.g. for the orthorhombic distortions in the orthoferrites). Nevertheless, even in such a case the exchange is perturbed for any orientation of the light polarization, only the amplitude may be slightly different and such differences will be very hard to distinguish experimentally. Indeed, the lattice constants along the *x, y* and *z* axes in the orthoferrites vary by a few per cent. At the same time the in plane cross-section of the unit-cell in the *z*-cut $FeBO_3$ and α-Fe_2O_3 has a hexagonal shape that also would give rise to a very slight if not negligible variation of the exchange perturbation due to the polarization of light.

Supplementary note 3 – Does light increase or decrease *D*/*J* in $ErFeO_3$?

The magnetocrystalline anisotropy of $TmFeO_3$ and $ErFeO_3$ is characterized by a strong temperature dependence in the ~ 80 – 100 K temperature interval [18]. In this temperature range the spin configuration of the iron sub-lattices continuously rotates in the (*xz*)-plane, while keeping the weak ferromagnetic moment in the same plane. Thus one might anticipate a strong temperature dependence of the THz emission in the vicinity of the spin-reorientation temperature interval. Indeed, along with the quasi-antiferromagnetic mode, another mode at ~ 100 GHz appears in the spectra of emission generated in $TmFeO_3$ and $ErFeO_3$ in vicinity of the spin-reorientation temperature range. We attribute this second mode to the quasi-ferromagnetic precession of spins.

The quasi-ferromagnetic resonance excitation under optical excitation of $TmFeO_3$ and $ErFeO_3$ samples near the spin reorientation temperature region has been reported before and assigned to the thermally induced change of the anisotropy [19-21]. This picture concurs with the fact that in $YFeO_3$ and $FeBO_3$ samples the low frequency mode has not been observed because of the absence of a spin reorientation in these materials.

The analysis of the waveforms generated in $TmFeO_3$ sample at the temperatures of the photo-induced spin reorientation allowed us to determine the absolute sign of the change of *D*/*J* as discussed in the main text of the paper. It is instructive to check whether the sign of the quasi-antiferromagnetic oscillations generated in another compound exhibiting spin reorientation, namely in the erbium orthoferrite $ErFeO_3$, is consistent with the obtained result. We applied the same analysis to the signals generated in the *x*-cut $ErFeO_3$ sample as illustrated in Supplementary Fig. 8. Importantly, in the latter sample the direction of the spin reorientation is opposite with respect to that in the *z*-cut crystalline plate of $TmFeO_3$. Thus, the initial phase of the quasi-antiferromagnetic mode must be the same as the initial phase of the low-frequency quasi-ferromagnetic mode [Supplementary Fig. 8 (a)]. This prediction has been fully validated by the experimental data, as shown in Supplementary Fig. 8 (b).

Supplementary note 4 – Macroscopic theory of the quasi-antiferromagnetic mode excitation via optical perturbation of the exchange interaction

The equilibrium orientation of the iron spins in canted antiferromagnets is given by the minimum of the thermodynamic potential[2]:

$$\Phi = J\mathbf{S}_1\mathbf{S}_2 + 2\mathbf{D}\cdot[\mathbf{S}_1\times\mathbf{S}_2] + K_x\left(S_{1x}^2 + S_{2x}^2\right) + K_y\left(S_{1y}^2 + S_{2y}^2\right) + K_z\left(S_{1z}^2 + S_{2z}^2\right) + \\ + K_4\left(S_{1x}^4 + S_{1y}^4 + S_{1z}^4 + S_{2x}^4 + S_{2y}^4 + S_{2z}^4\right), \quad (25)$$

where $\mathbf{S}_1$ and $\mathbf{S}_2$ are the vectors that characterize the spins of the iron ions in the two magnetic sublattices, J is the nearest-neighbor isotropic exchange interaction constant; $\mathbf{D}$ is a constant vector pointing along the y-axis and describing the Dzyaloshinskii-Moriya antisymmetric exchange interaction; K_x, K_y, K_z, K_4 are the constants of the effective anisotropy. The K_x, K_y, K_z, K_4 are purely phenomenological and "effective" parameters, in the sense that they do not necessarily have a clear physical meaning, being a combinations of the single ion anisotropy terms and "hidden" exchange coupling parameters (see Ref. 22 for a detailed discussion).

It is instructive to rewrite the potential (25) in terms of the ferromagnetic vector (magnetization) $\mathbf{M} = -\gamma(\mathbf{S}_1 + \mathbf{S}_2)$ and the antiferromagnetic vector $\mathbf{L} = -\gamma(\mathbf{S}_1 - \mathbf{S}_2)$ as follows [1]

$$\Phi = -JM_{\mathrm{Fe}^{3+}}^2 + \frac{1}{2}JM^2 + D[M_xL_z - M_zL_x] + \frac{1}{2}(K_y - K_x)L_y^2 + \frac{1}{2}(K_z - K_x)L_z^2 + \frac{1}{4}K_4L^4. \quad (26)$$

Here γ is the absolute value of the gyromagnetic ratio of an electron and $M_{\mathrm{Fe}^{+3}}$ is the magnetic moment of the iron ion. Note that $M^2 + L^2 = 4M_{\mathrm{Fe}^{+3}}^2$. Let us consider the x-cut $ErFeO_3$ or $YFeO_3$ as an example (the case of other materials is identical after rotation of the coordinate system by 90°). Taking into account that in canted antiferromagnets $J >> D >> K_x$, K_y, $K_z >> K_4$ [1], the equilibrium magnetic configuration reads

$$M_x = M_y = L_y = L_z = 0,\ L_x = L_0 \approx 2M_{\mathrm{Fe}^{+3}},\ M_z = M_0 \approx \frac{D}{J}L_0. \quad (27)$$

Here D/J defines the canting angle, which is a small parameter; in the subsequent derivation all terms smaller than the canting angle are neglected.

The spin dynamics is described by the Landau-Lifshitz equations for ferromagnetic and antiferromagnetic vectors

---

[2]The formulae in this file are written in the Gaussian system of units, but the final results are converted to SI units.

$$\frac{d\mathbf{M}}{dt}=\gamma\left(\left[\mathbf{M}\times\frac{\partial\Phi}{\partial\mathbf{M}}\right]+\left[\mathbf{L}\times\frac{\partial\Phi}{\partial\mathbf{L}}\right]\right),\quad \frac{d\mathbf{L}}{dt}=\gamma\left(\left[\mathbf{M}\times\frac{\partial\Phi}{\partial\mathbf{L}}\right]+\left[\mathbf{L}\times\frac{\partial\Phi}{\partial\mathbf{M}}\right]\right). \tag{28}$$

The vectors **M** and **L** can be represented as a sum of the equilibrium $\mathbf{M}_0$ and $\mathbf{L}_0$ and the time-varying **m**(t) and **l**(t) components. In the case of small deviations from equilibrium ($m \ll M_0$ and $l \ll L_0$) one can obtain a linearized system of equations describing the quasi-antiferromagnetic resonance:

$$\begin{cases}\dfrac{dm_z}{dt}=\gamma\left(K_y-K_x\right)L_0 l_y,\\ \dfrac{dl_x}{dt}=-\gamma\left(K_y-K_x\right)M_0 l_y,\\ \dfrac{dl_y}{dt}=-\gamma\left(JL_0+DM_0\right)m_z+\gamma DL_0 l_x.\end{cases}, \tag{29}$$

From the system (29) it follows that l_y obeys the harmonic oscillator equation

$$\frac{d^2 l_y}{dt^2}+\omega_{\mathrm{qAF}}^2 l_y=0, \tag{30}$$

where $\omega_{\mathrm{qAF}}\approx\gamma\sqrt{2H_{\mathrm{E}}H_{\mathrm{A}}}$ is the frequency of the quasi-antiferromagnetic mode, $H_{\mathrm{E}}=\frac{1}{2}JL_0$ is the exchange field and $H_{\mathrm{A}}=\left(K_y-K_x\right)L_0$ is the anisotropy field.

Eqs. (29) and (30) describe the free dynamics of the spins without damping. To include the light-induced stimulus due to the inverse isotropic magnetic refraction, one has to consider an additional contribution, proportional to the envelope of the optical intensity I_{opt}, $\Phi_{\mathrm{IMR}}=-\sum_{i,j} aI_{\mathrm{opt}}M^2$, added to the thermodynamic potential (25). As discussed above this term can be considered as a light-induced perturbation of the symmetric exchange energy, i.e.

$$\Phi_{\mathrm{ex}}=\Phi_{\mathrm{ex}\,0}+\Phi_{\mathrm{IMR}}=\frac{1}{2}J_0M^2-aI_{\mathrm{opt}}M^2=\frac{1}{2}\left(J_0+\Delta J\right)M^2, \tag{31}$$

where $\Delta J=-2aI_{\mathrm{opt}}$, and the coefficient a can be calculated from the microscopic theory of non-equilibrium exchange presented in the previous section. According to Eq. (24) the perturbation of the exchange $\Delta J(t)$ follows the envelope of the optical intensity, i.e. $\Delta J(t)=\Delta J_0\exp\left(-t^2/\tau^2\right)$, where ΔJ_0 is the peak change of the exchange parameter, $\tau=\tau_{\mathrm{FWHM}}/2\sqrt{\ln 2}$, τ_{FWHM} being the

full width at half maximum of the laser pulse intensity envelope. However, it is also possible that the antisymmetric Dzyaloshinskii-Moriya energy Φ_D is also modified such as

$$\Phi_{\mathrm{D}} = (D_0 + \Delta D)[M_x L_z - M_z L_x], \tag{32}$$

where ΔD is proportional to the envelope of the optical intensity, i.e. $\Delta \mathrm{D}(t) = \Delta D_0 \exp\left(-t^2/\tau^2\right)$. The light-induced modification of the exchange energy creates the torque $\sim \left[\mathbf{L}\times\frac{\partial\Phi}{\partial\mathbf{M}}\right]+\left[\mathbf{M}\times\frac{\partial\Phi}{\partial\mathbf{L}}\right]$ acting on the antiferromagnetic vector **L**. It appears on the right-hand side of the equation of motion of l_y as a driving force:

$$\frac{d^2 l_y}{dt^2} + \omega_{\mathrm{qAF}}^2 l_y = -\gamma L_0 M_0 \frac{d(\Delta J)}{dt} + \gamma\left(L_0^2 - M_0^2\right)\frac{d(\Delta D)}{dt}. \tag{33}$$

Note, that here we restrict ourselves to the terms of first order in smallness with respect to ΔJ, ΔD ,l_y, l_x and m_z. Moreover, since $L_0^2 >> M_0^2$ we can neglect the last term on the right side of Eq. (33). Let us also introduce some phenomenological damping into the equation of motion (33) as

$$\frac{d^2 l_y}{dt^2} + 2\nu\frac{dl_y}{dt} + \omega_{\mathrm{qAF}}^2 l_y = -\gamma L_0 M_0 \frac{d(\Delta J)}{dt} + \gamma L_0^2 \frac{d(\Delta D)}{dt}, \tag{34}$$

where ν is the damping parameter.

Applying the Fourier transformation to Eq. (34) with respect to time t we get

$$\omega^2\tilde{l}_y + 2\mathrm{i}\nu\omega\tilde{l}_y + \omega_{qAF}^2\tilde{l}_y = \gamma L_0\left(M_0\Delta J_0 \text{-} L_0\Delta D_0\right)\sqrt{\pi}\,\mathrm{i}\,\omega\tau\exp\left(-\frac{\omega^2\tau^2}{4}\right), \tag{35}$$

where $\tilde{l}_y(\omega)$ is a Fourier transform of $l_y(t)$

$$l_y(t) = \frac{1}{2\pi}\int_{-\infty}^{\infty} l_y(\omega)\mathrm{e}^{\mathrm{i}\omega t}d\omega. \tag{36}$$

By rearranging Eq. (35) we obtain

$$\tilde{l}_y = -\gamma L_0(M_0\Delta J_0 - L_0\Delta D_0)\frac{\mathrm{i}\omega}{\omega_{\mathrm{qAF}}^2 - \omega^2 + 2\mathrm{i}\nu\omega}\sqrt{\pi}\tau\exp\left(-\frac{\omega^2\tau^2}{4}\right). \tag{37}$$

At the same time, it follows from Eqs. (29) that $\mathrm{i}\,\omega\tilde{m}_z = \gamma L_0 \left(K_y - K_x\right)\tilde{l}_y$, where $\tilde{m}_z$ is a Fourier transform of m_z. Thus, using Eq. (37) and the relations $\gamma^2 L_0^2 \left(K_y - K_x\right) = \frac{\omega_{\mathrm{qAF}}^2}{J_0}$ and $\frac{M_0}{L_0} = \frac{D_0}{J_0}$ we can write

$$\tilde{m}_z = -\left(\frac{\Delta J_0}{J_0} - \frac{\Delta D_0}{D_0}\right)\omega_{\mathrm{qAF}}^2 M_0 \frac{1}{\omega_{\mathrm{qAF}}^2 - \omega^2 + 2\mathrm{i}\nu\omega}\sqrt{\pi}\tau \exp\left(-\frac{\omega^2\tau^2}{4}\right). \tag{38}$$

It is instructive to consider the limit case of the instantaneous torque, i.e. $\tau\ \omega_{\mathrm{qAF}} << 2\pi$. In this case Eq. (38) can be approximated as

$$\tilde{m}_z \approx -\left(\frac{\Delta J_0}{J_0} - \frac{\Delta D_0}{D_0}\right)\omega_{\mathrm{qAF}}^2 M_0 \frac{1}{\omega_{\mathrm{qAF}}^2 - \omega^2 + 2\mathrm{i}\nu\omega}\sqrt{\pi}\tau\ . \tag{39}$$

By calculating the inverse Fourier transform of Eq. (38) we can get an analytical solution for the magnetization response (in the case of small damping $\nu << \omega_{\mathrm{qAF}}$)

$$m_z(t) = \Theta(t) m_0 \sin(\omega_{\mathrm{qAF}} t) e^{-\nu t}\ , \tag{40}$$

where $\Theta(t)$ is Heaviside function and

$$m_0 = -\left(\frac{\Delta J_0}{J_0} - \frac{\Delta D_0}{D_0}\right)\sqrt{\pi}\omega_{\mathrm{qAF}}\tau M_0\ . \tag{41}$$

Eq. (41) can be rewritten in the form

$$m_0 = -\left[\Delta\left(\frac{D}{J}\right)\Big/\left(\frac{D_0}{J_0}\right)\right]\sqrt{\pi}\omega_{\mathrm{qAF}}\tau M_0\ , \tag{42}$$

where

$$\Delta\left(\frac{D}{J}\right) = \frac{D_0 - \Delta D_0}{J_0 - \Delta J_0} - \frac{D_0}{J_0}\ . \tag{43}$$

Eq. (42) shows that the ratio between the magnetization deviation from equilibrium m_0 and the spontaneous magnetization M_0 is directly proportional to the relative change of the ratio $\frac{D}{J}$.

It follows from the Eq. (26) that a light-induced change of the out of plane anisotropy $\left(K_y - K_x\right)$ cannot trigger the quasi-antiferromagnetic mode since in equilibrium there is no spin alignment along the y-axis and $L_y = 0$. K_z is not involved in the quasi-antiferromagnetic excitation at all [e.g. it is absent in Eqs. (29)].

Generally speaking, the change of anisotropy must trigger the quasi-ferromagnetic mode [20]. At the same time, the excitation of this low-frequency mode has been observed only in $TmFeO_3$ and $ErFeO_3$ and then only near the temperature region of the phase transition (see Supplementary note 2). At these temperatures one indeed expects a significant thermally induced change of the anisotropy. This observation unambiguously proves that, with the exception of the spin-reorientation temperature interval in $TmFeO_3$ and $ErFeO_3$, the contribution of laser-induced anisotropy changes to the observed photo-excited dynamics is negligible. In the case of $FeBO_3$ and α-Fe_2O_3 the situation is even more obvious since these materials are easy-plane antiferromagnets with negligibly small in-plane anisotropies.

Supplementary note 5 – Electrodynamics of THz generation and emission in canted antiferromagnets

The optical pulse excites the magnetization oscillations in a thin slab of a canted antiferromagnet. The lateral dimensions of the sample and the size of the excitation spot (several mm) are much larger than the THz wavelength (300 μm). Thus we can restrict ourselves to a one-dimensional problem as follows.

Let us consider an infinite slab of a material of thickness d with the permittivity ε_S, containing an oscillating magnetization **M** oriented along the z-axis in the form

$$\mathbf{M}(t,x) = (M_0 + m(t))[\Theta(x+d) \quad \Theta(x)]\mathbf{z}_0, \tag{44}$$

where M_0 is the equilibrium magnetization, m(t) is the time-dependent deviation from equilibrium, and $\Theta(x)$ is the Heaviside function. The magnetic permeability in the canted antiferromagnets in the vicinity of the magnetic resonance frequency varies from 0.9 to 1.1 [1], and thus can be neglected. From the physical point of view, it means that the inverse action of the emitted magnetic field on the oscillating magnetization is negligible.

To find the emission of the magnetization (44) one has to solve the wave equation for the y-component of the electric field E_y

$$\frac{\partial^2 E_y}{\partial x^2} - \frac{\varepsilon}{c^2}\frac{\partial^2 E_y}{\partial t^2} = \frac{4\pi}{c^2}\frac{\partial j}{\partial t}, \tag{45}$$

where $j = c\frac{\partial M}{\partial x} = cm(t)[\delta(x+d) - \delta(x)]$, $\delta(x)$is a Dirac function and the permittivity $\varepsilon(x)$ in the THz range is ε_s in the slab ($-d < x < 0$) and unity outside, respectively. Eq. (45) is derived from the Maxwell equations.

After applying the Fourier transformation with respect to time, Eq. (45) transforms to

$$\frac{\partial^2 \tilde{E}}{\partial x^2} + \frac{\varepsilon(x)\omega^2}{c^2}\tilde{E} = \frac{4\pi}{c}\mathrm{i}\,\omega\tilde{m}(\omega)[\delta(x) - \delta(x+d)]. \tag{46}$$

We solve Eq. (46) in the homogeneous regions $x < -d$, $-d < x < 0$ and $x > 0$ and match the solutions by the boundary conditions that arise after integrating Eq. (46) across the boundary at $x = -d$ and $x = 0$. These boundary conditions imply the continuity of $\tilde{E}$, while $\frac{\partial \tilde{E}}{\partial x}$ exhibits a

finite discontinuity of $\frac{4\pi}{c} i\omega\tilde{m}(\omega)$ and $-\frac{4\pi}{c} i\omega\tilde{m}(\omega)$ at the boundaries $x =$ $-d$ and $x = 0$ respectively. The solution has the form

$$\tilde{E} = \begin{cases} R\mathrm{e}^{-\mathrm{i}k_0 x}, x > 0 \\ F\,\mathrm{e}^{-\mathrm{i}kx} + B e^{\mathrm{i}kx}, \quad d < x < 0, \\ C\,\mathrm{e}^{\mathrm{i}k_0(x+d)}, x < \quad d \end{cases} \tag{47}$$

where $k_0 = \frac{\omega}{c}$ is the wavenumber in free space, $k = \frac{\omega}{c}\sqrt{\varepsilon_s} = \frac{\omega}{c} n$ is the wavenumber in the slab with refractive index n. In our experiment we measured the field $E(t)$ emitted into free space given by

$$E(t) = \frac{1}{2\pi}\int_{-\infty}^{\infty} R(\omega)\mathrm{e}^{\mathrm{i}\omega t}\, d\omega, \tag{48}$$

where

$$R(\omega) = \frac{4\pi\omega}{c}\tilde{m}(\omega)\frac{2k + (k_0 - k)e^{-\mathrm{i}kd} - (k_0 + k)e^{\mathrm{i}kd}}{(k - k_0)^2 e^{-\mathrm{i}kd} - (k - k_0)^2 e^{\mathrm{i}kd}}\,. \tag{49}$$

The function $\tilde{m}(\omega)$ has been already derived above and is given by Eq. (38).

The refractive index at the quasi-antiferromagnetic frequency equals n = 4.71 and 4.68 for $TmFeO_3$ and $YFeO_3$, respectively [1]. The thickness of our samples equals d = 60 μm and 70 μm correspondingly. By substituting these parameters into Eqs. (48) and (49) and fitting the amplitude, the time delay corresponding to the arrival of the pump pulse and the damping constant in Eq. (38), we obtained the waveforms shown in Supplementary Fig. 9 for different durations of the excitation torque. It is seen in the figure that the waveforms calculated for the excitation torque with a duration of < 0.5 ps agree well with the experimental data. As the duration of the torque exceeds 0.5 ps the calculated waveforms become different from the experimental signals because the spins start to follow the torque adiabatically, leading to the generation of a broadband electric field pulse not observed in the measurements. When the rise-time becomes longer than 1 ps the excitation of the quasi-antiferromagnetic mode gradually vanishes since the spectrum of the torque does not overlap with the resonance frequency

anymore. Importantly, to keep the excited quasi-antiferromagnetic oscillation of the same amplitude one has to increase the peak amplitude of the torque as its duration becomes longer.

However, even for the case of the shortest torque (100 fs) both calculated and measured signals demonstrate a finite rise-time (~ 1 ps). This effect is due to the fact that the samples act as Fabry-Perot resonators for the electromagnetic radiation at the frequencies of interest. The Fabry-Perot behavior is described by the term $\frac{2k+(k_0-k)e^{-\mathrm{i}kd}-(k_0+k)e^{\mathrm{i}kd}}{(k-k_0)^2 e^{-\mathrm{i}kd}-(k-k_0)^2 e^{\mathrm{i}kd}}$ in Eq. (49). The electric field emitted by the instantaneously commencing magnetization oscillations does not follow the magnetization immediately but exhibits a finite rise time determined by the characteristic timescale of the resonator $\frac{nd}{c}$ ~1 ps. The presence of Fabry-Perot resonances in the vicinity of quasi-antiferromagnetic frequency was also experimentally verified in our THz absorption measurements.

In $FeBO_3$ and α-Fe_2O_3 the period of quasi-antiferromagnetioc oscillation is several times smaller than in the orthoferrites. Therefore, excitation is possible for longer torques. However, it is natural to assume that the mechanism of excitation and its timescale are the same in these materials as in the orthoferrites.

Using Eq. (49) and measured values of the electric field we estimated the amplitude of the oscillating magnetization as ~10^{-3}emu/cm^3in the orthoferrites and the iron borate (~1 A/m in SI units), which implies $\Delta\left(\frac{D}{A}\right)/\left(\frac{D_0}{A_0}\right) \approx 0.01$ %. The amplitude of the electric field generated in the hematite is smaller, but this material absorbs light at 800 nm, which leads to a different regime of the excitation. Thus, the radiated emission might have been generated in a thin surface layer in which the optical pulse penetrates, which would lead to a similar efficiency of the perturbation of the exchange energy.

Supplementary note 6 – The strength of the optical control of the super-exchange

Even a relatively small change of the exchange interaction corresponds to rather large effects expressed in absolute units of equivalent field and energy.

From Eq. (33) one can see that the changing of the ratio D/J is equivalent to the application of the short pulsed magnetic field $\mathbf{b}_{\mathrm{eff}}$ along the magnetization direction. The peak amplitude of this field is

$$b_0 = \left(\Delta J_0 M_0 - \Delta D_0 L_0\right) = \frac{J_0 m_0}{\sqrt{\pi}\omega_{\mathrm{qAF}}\tau}. \qquad (50)$$

For the parameters of our experiment and the known strength of the exchange field in the materials under study ($J_0 L_0$ ~ 1000 Tesla) we estimate the amplitude of the equivalent field to be of the order of ~ 0.01 Tesla per 1 mJ/cm^2 pump fluence. This value is of the same order of magnitude as the maximal strength of the light-induced magnetic field (per the same pump fluence) achieved with help of opto-magnetic phenomena such as the inverse Faraday effect [23]. However, the inverse Faraday effect and similar phenomena owe their strength to the spin-orbit coupling and therefore are not strong in all magnetic materials. Therefore the exchange driven optical control of spins should be a more versatile tool for the manipulation of magnetic states. The short single-cycle THz pulses of magnetic field which are shown to excite spin dynamics can achieve a peak amplitude of 0.1 Tesla [24]. Unfortunately, to generate such a pulse one needs extremely high pump fluences and dedicated laser systems not widely available. Indeed, the pulses of magnetic field used in Ref. 24 were generated using optical pulses with energy of 5 mJ, that is larger than the total energy of pulses generated in a conventional amplified Ti:sapphire laser used in our measurements.

The energy of the interaction between light and magnetic system can be estimated as

$$\Delta W = V b_0 M_0, \qquad (51)$$

where V is the optically excited volume of the material (~100 μm×1 mm×1 mm in our measurements). Using Eq. (50) and taking $M_0 \approx 10$ emu/cm^3 we estimate the energy ΔW is of the order of 1 μJ per excited area of 1 cm^2.

Finally we note that the 0.01% change of the ratio of the two exchange parametrs represents a difference of the relative changes of each of them. This means that if the changes are

of the same sign (which is quite likely) then each of them could in fact be much greater than 0.01% in agreement with the prediction of the microscopic theory.

Supplementary references

1. Srinivasan, G. (Ed.), Slavin, A. N. (Ed.). *High frequency processes in magnetic materials* (World Scientific Publishing, Singapore, 1995).

2. Koshizuka, N., Hayashi, K. Temperature dependences of one-magnon light scattering in $RFeO_3$. *J. Magn. Magn. Mater*. **31**, 569 (1983).

3. Chou, S. G., *et al*. High-resolution terahertz optical absorption study of the antiferromagnetic resonance transition in hematite (α-Fe_2O_3). *J. Phys. Chem. C* **116**, 16161 (2012).

4. Secchi A., Brener, S., Lichtenstein, A. I., Katsnelson, M. I. Non-equilibrium magnetic interactions in strongly correlated systems. *Annals of Physics* **333,** 221 (2013).

5. Schwinger, J. Brownian motion of a quantum oscillator. *J. Math. Phys*. **2**, 407 (1961).

6. Keldysh, L. V. Diagram technique for nonequilibrium processes. *Zh. Eksp. Teor. Fiz.* **47**, 1515 (1964); *Sov. Phys. JETP* **20**, 1018 (1965).

7. Kadanoff, L. P. & Baym, G. *Quantum Statistical Mechanics* (W. A. Benjamin, Inc. New York, 1962).

8. Katsnelson, M. I. & Lichtenstein, A. I. First-principles calculations of magnetic interactions in correlated systems. *Phys. Rev. B* **61**, 8906 (2000).

9. Mattis, D. C. *The Theory of Magnetism Made Simple* (World Scientific, Singapore, 2006).

10. Koch, E. Exchange mechanisms, Modeling and Simulation Vol. 2, Eds. Pavarini, E., Koch, E., Anders, F., and Jarrell, M. Forschungszentrum Jülich, 2012.

11. Kohn, W. Theory of Bloch electrons in a magnetic field: the effective hamiltonian. *Phys. Rev*. **115**, 1460 (1959).

12. Luttinger, J. M. The effect of a magnetic field on electrons in a periodic potential. *Phys. Rev*. **84**, 814 (1951).

13. Peierls, R. On the theory of diamagnetism of conduction electrons. *Z. Phys*. **80**, 763 (1933).

14. Grifoni, M. & Hänggi, P. Driven quantum tunneling. *Phys. Rep.* **304**, 229 (1998).

15. Dittrich, T., Hänggi, P., Ingold, G. L., Kramer, B., Schön, G., & Zwerger, W. *Quantum Transport and Dissipation* (Wiley-VCH, Weinheim, 1998).

16. Dunlap, D. H. & Kenkre, V.M. Dynamic localization of a charged particle moving under the influence of an electric field. *Phys. Rev. B* **34**, 3625 (1986).

17. Grossmann, F., Dittrich, T., Jung, P. & Hänggi, P. Coherent destruction of tunneling. *Phys. Rev. Lett*. **67**, 516 (1991).

18. White, R. L. Review of recent work on the magnetic and spectroscopic properties of the rare-earth orthoferrites. *J. Appl. Phys*. **40,** 1061 (1969).

19. Kimel, A. V., Kirilyuk, A., Tsvetkov, A., Pisarev, R. V. & Rasing, Th. Laser-induced ultrafast spin reorientation in the antiferromagnet $TmFeO_3$. *Nature* **429,** 850 (2004).
20. Kimel, A. V. *et al*. Optical excitation of antiferromagnetic resonance in $TmFeO_3$. *Phys. Rev. B* **74,** 060403 R (2006).
21. Jong, J. A. de, Kimel, A. V., Pisarev, R. V., Kirilyuk, A., & Rasing, Th. Laser-induced ultrafast spin dynamics in $ErFeO_3$. *Phys. Rev. B* **84**, 104421 (2011).
22. Herrmann, G. F. Magnetic resonances and susceptibility in orthoferrites. *Phys. Rev*. **133**, A1334 (1964).
23. Kimel, A. V. *et al*. Ultrafast non-thermal control of magnetization by instantaneous photomagnetic pulses. *Nature* 435, 655 (2005).
24. Kampfrath, T. *et al*. Coherent terahertz control of antiferromagnetic spin waves. *Nature Photonics* **5,** 31 (2011).
